# Validation of a cross-NTD toolkit for assessment of NTD-related morbidity and disability. A cross-cultural qualitative validation of study instruments in Colombia

**DOI:** 10.1371/journal.pone.0223042

**Published:** 2019-12-03

**Authors:** Janneke Fischer, Benita Jansen, Alberto Rivera, Libardo J. Gómez, Martha C. Barbosa, Jorge L. Bilbao, José M. González, Luis Restrepo, Yesenia Vidal, Ruth M. H. Peters, Wim H. van Brakel

**Affiliations:** 1 Athena Institute, Faculty of Science, VU University Amsterdam, Amsterdam, Netherlands; 2 Facultad Ciencias de la Salud, Universidad Metropolitana, Barranquilla, Colombia; 3 America de Sur, DAHW Deutsche Lepra- und Tuberkulosehilfe, Bogota, Colombia; 4 NLR, Amsterdam, Netherlands; Sciensano, BELGIUM

## Abstract

**Background:**

Many neglected tropical diseases (NTDs) are not fatal, but they are disabling, disfiguring and stigmatizing. More accurate data on these aspects would benefit planning, monitoring and evaluation of interventions, as well as provision of appropriate services for the often life-long consequences. In 2015, a cross-NTD toolkit was developed, consisting of a variety of existing questionnaires to measure morbidity, disability and health-related quality of life. The toolkit covers the domains of the International Classification of Functioning, Disability and Health (ICF) framework. These tools have been developed in a source country, however, it was intended for the cross-NTD toolkit to be applicable across NTDs in many countries with different cultures and languages in order to generate universally comparative data. Therefore; the present study aimed to validate several tools of the toolkit among people affected by leprosy or leishmaniasis in the cultural settings of Cartagena and Cúcuta, Colombia.

**Methodology:**

This study aimed to validate the following tools among 55 participants between 18–85 years old, affected by leprosy and leishmaniasis: (I) Clinical Profile, (II) Self-Reporting Questionnaire (SRQ), (III) WHO Quality of Life assessment-abbreviated version (WHOQOL-BREF), and (IV) WHO Quality of Life assessment-Disability (WHOQOL-DIS). The tools were administered during face-to-face interviews and were followed by open questions about the respondents’ thoughts on format of the tool and the understanding, relevance and acceptability of the items. The tools were validated using a qualitative method approach based on the framework for cultural equivalence, measured by the cultural, item, semantic and operational equivalences.

**Results:**

The Clinical Profile was seen as acceptable and relevant, only the semantic equivalence was not as satisfying and needs a few adaptations. The SRQ was very well understood and shows to reach the equivalences for the population of Colombia without any additional changes. Several items of the WHOQOL-BREF and the WHOQOL-DIS were not well understood and changes are recommended due to semantic difficulties. Operational equivalence of both questionnaires was not as desired in relation to the used response scales. The participants shared that the tools are relevant and important for their particular situation.

**Conclusions/Significance:**

The SRQ is found to be a valid tool for Colombia and can be included in the cross-NTD toolkit. The Clinical Profile, WHOQOL-BREF & WHOQOL-DIS need changes and retesting among Colombian people affected by an NTD. The toolkit as a whole is seen as useful to show the effects leprosy and leishmaniasis have on the participants. This cultural validation will contribute to a universally applicable cross-NTD toolkit.

## Introduction

Neglected tropical diseases (NTDs) are a group of diverse infectious diseases with high impact on morbidity and mortality rates in developing countries [1]. The pathogens responsible for NTDs are viruses, bacteria and parasites [2]. The WHO composed a list of 20 prioritized NTDs, which include diseases as helminthiases, Chagas disease, leprosy and leishmaniasis [3–5]. Annually, NTDs cause 534,000 deaths [6]. However, most NTDs are not fatal, but are disabling, disfiguring and stigmatizing [7]. For example, soil-transmitted helminths (STH) can impair children’s physical and cognitive development [7], and leprosy and human African trypanosomiasis (HAT) can cause neuro-disability [8, 9]. At societal level, NTDs can cause economic losses due to a loss of productivity, high costs for health care for households and thus increased poverty. Given the high prevalence and the impact of these diseases, the battle against NTDs and their consequences is still insufficient in scope.

Several research projects and healthcare institutes aim to map the prevalence and mortality of NTDs. In addition, data is needed on the impact of NTDs on affected persons, their families and society [6]. There is a lack of data on the morbidity and disabling consequences of NTDs. This hampers advocacy for interventions and funders. Furthermore, the planning, monitoring and evaluation of the interventions are more efficient if more accurate data would be available. Also, appropriate services for the often life-long consequences could be established if this gap would be fulfilled. Because NTDs disable rather than kill, various tools have been developed or adapted to measure the disabling consequences, for example by the use of disability-adjusted life years (DALY) [10–15]. These tools include health-related quality of life (QOL) questionnaires. To date, several different QOL questionnaires have been used, which makes results difficult to compare. This is also true for other aspects of NTD-related disability, stigma and social participation and related issues. To date, there is no agreed cross-NTD toolkit to assess and monitor the different disabling aspects of multiple NTDs.

In February 2015, the development of a cross-NTD toolkit was identified as an urgent need by the participants (scientists, experts and practitioners from different NTD backgrounds) of the ‘NTD Cross-cutting Issues Workshop’ in Utrecht, Netherlands [16]. This affirmed preceding recommendations of the Neglected Tropical Disease Non-Governmental Development Organization Network (NNN) who stated”the development of a generic cross-NTD toolkit to assess and monitor NTD-related morbidity and disability is one of the key priorities in the NTD field” [16]. The cross-NTD toolkit will enable the collection of internationally comparable data on disability caused by multiple NTDs and fulfil the existing gap [12, 17]. Such data can be used as a baseline for intervention strategies and provide useful information to identify priority areas for disease management, disability and inclusion (DMDI), which is a more extended version of ‘morbidity management and disability prevention’ (MMDP).

In August 2015, a cross-NTD toolkit was developed by Van ‘t Noordende and colleagues at Netherlands Leprosy Relief (NLR) and the VU University Amsterdam in collaboration with Neglected Tropical Disease NGDO network [18]. The development of the toolkit was based on consensus of a Delphi panel of NTD experts and a subsequent literature review on the validity of these instruments. The initial validation of the toolkit has been done in Fortaleza, Brazil, among people with Chagas disease, leishmaniasis, leprosy and schistosomiasis[18]. Since the cross-NTD toolkit needs to be applicable in cross-cultural settings, validation of the toolkit in various countries is required. Health-related concepts such as quality of life, disability and participation may differ across cultures [19]. While the WHOQOL-BREF and the SRQ have been widely validated, this was often not among poor, low-literate or illiterate target groups, who are typically the people affected by NTDs. The WHOQOL-DIS has been validated and used much less often. We therefore consider important to validate tools that are used to measure such concepts in a cross-cultural design [20–23]. In case the tools to measure the concepts are not reliable in a given context, the results and conclusions drawn can be incorrect.

This study validated the following tools of the cross-NTD toolkit in the cultural setting in Colombia: (I) Clinical Profile [18], (II) Self-Reporting Questionnaire (SRQ) [24], (III) WHO Quality of Life assessment-abbreviated version (WHOQOL-BREF) [25], and (IV) WHO Quality of Life assessment-Disability (WHOQOL-DIS) [26]. The tools were validated among people with leprosy and leishmaniasis in Cartagena and Cúcuta, Colombia.

## Methods

Ethical clearance from the ethics committee of the University Metropolitana of Barranquilla, by means internal act No. 033 of July1, 2016, was applied for and obtained before initiation of the data collection.

### Study design

This study is an evaluation of four equivalences of cultural validity of health related quality of life questionnaires assessing disability in NTD-affected adult population in Colombia. This was done in a cross-sectional manner among individuals affected by leprosy or leishmaniasis living in Cartagena and Cúcuta, Colombia within a period of 2 months. This study used a qualitative method approach.

#### Study sample

Study participants were selected through purposive sampling fulfilling a number of eligibility criteria. The study participants were of either sex, 18 years and above, had at least one of the clinical features of leprosy or leishmaniasis, and had the ability to answer questions of structured questionnaires during an interview. The identification and selection of potential study participants was facilitated through the use of patient registers in databases, which were provided by programs for leprosy and vector-transmittable diseases (Enfermedaded Transmitidas por Vectores, ETV), the health institute department of Norte de Santander in Cúcuta and the administrative health department of Cartagena (DADIS) and contacted via their caregivers. To obtain data saturation, the study aimed to include a minimum of 50 participants [27]. Eventually 55 participants were enrolled affected by leprosy (35 participants) or leishmaniasis (20 participants), of whom 56% were men and 44% women.

#### Tools included in this study

In this study the tools (I) Clinical Profile, (II) SRQ and (III & IV) WHOQOL-BREF & WHOQOL-DIS were validated. The tools of the toolkit cover domains of the International Classification of Functioning, Disability and Health (ICF) framework, developed by the WHO. The SRQ, WHOQOL-BREF & -DIS are validated instruments from official sources (WHO), while the Clinical profile was developed to complement the toolkit.

The Clinical Profile measures the domain ‘Body functions and structures’ (S1 and S2 Appendices) [28]. The questionnaire contains 20 questions with multiple level answers which provide no quantitative data, but rather highlight the problems the patients experience due to problems with body structure and functions [18]. The Clinical Profile was developed by Van ‘t Noordende *et al*. in 2015 to complete the toolkit, because there was no suitable existing short tool to assess body function and structure. This questionnaire was based on the ICF checklist, discussion with experts and a literature study. The tool was developed in the Netherlands and written in English. The questionnaire was translated into Brazilian Portuguese and pilot tested in the Ceará State of Brazil.

The SRQ measures the mental health aspect of the ICF domain ‘Body functions and structures’ (S3 and S4 Appendices)[29]. It has been developed by the WHO with the aim to screen for psychiatric disturbances, including depression, anxiety-related disorders and somatoform disorders as a first screening instrument [24]. The SRQ has especially been developed for the use in developing countries and contains 20 ‘yes/no’ questions, which can be self-administered or interviewer-administered [30]. The SRQ was originally developed in English and was translated into 20 languages, including Spanish. Validity studies show a high specificity and sensitivity for detecting mental disorders [31]. The SRQ-20 has previously been used in Colombia [32–34]. The Ministry of Health and the Department of Science, Technology and Innovations (a Colombian government agency) used this tool for their research among 13,200 people in 2015 [35].

The WHOQOL measures the perception of the participants regarding their quality of life in the context of their culture and values, their goals, standards and concerns [36]. The WHOQOL was simultaneously and collaboratively developed in 15 different centres worldwide from 14 developed and developing countries, using 12 languages [37]. The WHOQOL-BREF is a shorter version (26 questions), addressing the four quality of life domains physical health, psychological health, social relationships and environment (S5 and S6 Appendices) [38]. This questionnaire has been validated cross-culturally with a high validity, internal consistency and test-retest reliability [25]. The WHOQOL-BREF has been translated into Spanish and has been used in many Spanish speaking countries, including Colombia [39, 40]. In Colombia, this questionnaire has been used among individuals attending the private practice of a bariatric surgeon from Medellín, people affected by HIV/AIDS in Medellín and women with breast cancer in Antioquia [41–43]. This study took place in another part of Colombia focusing on a different target population; individuals affected by two of the listed NTDs.

The WHOQOL-DIS is a module that can be added to the WHOQOL-100 or WHOQOL-BREF, which was especially developed to measure the quality of life of adults with physical or intellectual disabilities [26]. This module has been developed in collaboration among 12 centers around the world to identify gaps in the coverage of the WHOQOL-100 and WHOQOL-BREF [26]. This is an additional domain of 12 items, also translated into Spanish. The Spanish version has been validated positively in Spain among persons with neurodegenerative disorders [44]. The WHOQOL-BREF and WHOQOL-DIS cover the domain ‘Personal factors’ of the ICF model (S5 and S6 Appendices) [45]. This questionnaire was only administered when question 18 of the Clinical Profile, “Do you have an impairment (disability/limitation)?” was answered with “yes”.

#### Testing four equivalences of cultural validity

In 1998 Herdman and colleagues published a study providing a framework for assessing the cultural equivalence of health-related quality of life (HRQoL) questionnaires [23]. The framework consists of five categories of equivalence: conceptual, item, semantic, operational and measurement equivalence. Various studies have shown that the framework of equivalence is a useful tool to validate tools cross-culturally, also for e.g. stigma and social participation [20, 46–49].

‘*Conceptual equivalence*’ refers to how a concept, for example ‘health and quality of life’ or physical and mental impairments is conceptualized. All domains assessed in the instrument should be equally relevant and important in the target culture as in the original culture [23]. The conceptual equivalence is achieved when the questionnaire has the same relationship to the underlying concept in both cultures, primarily in terms of the domains included and the emphasis placed on different domains assessed in the questionnaire. In the present study, the cultural equivalence was investigated by a literature research for questionnaires dealing with similar or related topics. Furthermore, the participants were asked about their thoughts on the importance and relevance of the domains related to the concept assessed with the questionnaire [23].

*‘Item equivalence’* refers to how the domains are conceptualized and sampled, and whether items are equally relevant and acceptable in the target culture and in the culture where the tool has been developed. The relevance of items may vary across cultures. For example, a question about skiing is not relevant in a country without snow. Also, questions can be offensive or may deal with taboo subjects [46]. The item equivalence was measured in this study during the cognitive interview that followed the administration of the tools as well in the group discussions. The participants were asked about their thoughts regarding the relevance and acceptability of the items used in the questionnaire. In addition, the participants’ responses were observed during the application of the questionnaire for nonverbal communication, e.g. facial expressions, hand gestures, tone and speed of talking[50].

‘*Semantic equivalence*’ refers to the understanding of the language used. It concerns the transfer of meaning across languages and the achievement of a similar effect on respondents in different languages [23]. This can be for example about using the appropriate level of a language. Experts in the field might use different words to describe a concept compared to the words the general public or lower educated persons would use. The semantic equivalence in this study was measured by observations of the participants during the administration of the questionnaires. Furthermore, the participants were asked about their understanding of the items and their thoughts about the level of Spanish used in these translations of the questionnaires. In addition, the opinion of Colombian bilingual experts in the field was gathered during a group discussion.

‘*Operational equivalence’* refers to the methods used to measure responses and whether they are appropriate in the target culture. For example, whether the questionnaire format is suitable, if the instructions are clear and whether the mode of administration is appropriate in the culture. In some cultures it is useful to ask open questions, while in other cultures closed questions will be preferred [51]. Another important factor is the reference to a time frame (e.g. when a participant is asked to think about the previous week or month). Some cultures do not differentiate between past, present and future, and a question about the previous week can only be asked about in terms of events that have happened that day [23]. The operational equivalence was attained if these elements did not influence the results.

As developed by Herdman *et al*. (1998), the fifth equivalence is ‘*Measurement equivalence’*. This refers to the equivalence of the outcome of the process, measuring the instruments’ behaviour. This can be the construct validity, internal consistency, reproducibility including agreement and reliability, floor and ceiling effects interpretability of the tool [20, 21]. For the measurement of this category, data of a quantitative study would be necessary.

The categories covered in this study are ‘*Conceptual equivalence’*, *Item equivalence’*, *‘Semantic equivalence’* and *‘Operational equivalence’* and therefore qualitative data was collected. Previous studies have shown that the qualitative methods were able to identify significant problems in wording of items of a questionnaire, which could not be identified by psychometric validation measurements. All five categories of equivalence must be achieved for an instrument to be ‘Culturally equivalent’ according to the framework of Herdman *et al*. [23, 49]. For this study, the tools were considered ‘valid’ for the use of the toolkit when the four qualitative equivalences tested were achieved [50, 52, 53]. However, the value of the quantitative measurement equivalence is recognized and recommended for future studies.

#### Translation

Three of the four tools that were validated in this study, SRQ, WHOQOL-BREF and WHOQOL-DIS had already been translated to Spanish according to strict WHO guidelines. However, the Clinical Profile and the interview guide group discussion guides were not. The Clinical Profile is available in English and Portuguese. Because this research was based on the pilot study in Brazil, the DAHW Colombia translated the exact same questionnaires used in the pilot study from Portuguese to Spanish. The translation was done by native Spanish speakers from Colombia who also understand the Portuguese language. This was translated back to Portuguese to ensure right transfer of meaning. The translation was conceptual and not literal, which aimed to be understandable for all Spanish-speaking Colombian people. All questionnaires had been tested on the colleagues of the DAHW. Subsequently, only a few words have been changed to facilitate linguistic understanding of the patients; without affecting in any way the content of the questions. The final versions of the instruments were tested in Barranquilla with the students of the Universidad Metropolitana before using them in this validation study.

### Data collection

This study used qualitative methods. The tools to be validated are quantitative questionnaires containing closed questions. However, this study focused on how the participants experienced answering the questionnaires. During cognitive interviews and group discussions following the administration of the questionnaires, the participants were asked about their thoughts on the relevance and the understanding of the tools.

### Interview

All tools were tested for each participant in the study. Two native Spanish-speaking Colombian students of Medicine at the Universidad de Metropolitana, Barranquilla, conducted the 55 interviews, supported by a student from the VU University of Amsterdam who recorded the interviews and wrote field notes. All were trained at the University before starting the actual interviews. The tools were administered during face-to-face interviews. Additional to each instrument, cognitive interviews were done with participants about their understanding of the tools and about their thoughts regarding the relevance of the instruments for their particular condition.

### Focus group discussions

Four focus group discussions were conducted to obtain more in-depth insights of the experiences the participants had with the tools. The groups were composed of 6–12 participant affected by leprosy (Cartagena), or leprosy or leishmaniasis (Cúcuta) selected by convenience sampling. The age range was between 19 and 68 years old, representative for both genders. The focus group discussions were led by Colombian health care professionals and the duration was approximately 60 to 90 minutes. The topics of these group discussions were the experience of the participants with the tools and their experiences of living in Colombia with an NTD (S7 and S8 Appendices). Furthermore, there has been a group discussion between the researchers about their experiences and about possible explanations for the results and their interpretation of the present study.

### Observations per item

During the administration of the questionnaires notes were made for each question. A tick mark (✓) was given if a question was directly understood, a cross (✗) if a question needed to be reformulated before the question was understood, and a zero (0) if examples needed to be given before a question was understood.

### Analysis

The cognitive interviews were recorded, transcribed and translated into English. All data was entered using Excel 2010 for Mac and the transcribed interviews were analysed using the qualitative analysis software MAXQDA 12. Open coding was used to measure the recurring opinions of participants related to the equivalences. Conceptual equivalence is analysed by coding for statements about importance and relevance of the concepts, as well as existing literature on the concepts in the target culture. Item equivalence is analysed by coding for statements about relevance and acceptability of the items, as well as observations of the responses given. Semantic equivalence is analysed by coding for terms about understanding of the items, opinions about words used and level of Spanish. In addition this equivalence is measured by observations of the participants during the administration of the questionnaires. Operational equivalence is analysed by coding for statements about timeframes, response options, duration, layout and form of administration. In addition for all equivalences, the opinion of Colombian bilingual experts in the field was gathered during a group discussion. All interviews were analysed in a systematic manner for reoccurring statements and quotes are provided. When 20% of the participants did not understand a question without additional rephrasing or examples, we interpreted them as not well understood in order to facilitate interpretation of the results.

### Ethical considerations

Patient data was extracted from databases of programmes for leprosy and leishmaniasis. This contained data on home addresses and telephone numbers of patients, which were used to contact them and invite them to participate in the study. This data was not used for other purposes and was not accessible to unauthorized parties. The participants were fully informed about the nature and objective of the study and on the confidentiality of the data. Before the interview the participants voluntarily agreed to participate by signing a written informed consent form (S9 Appendix). As mentioned, ethical clearance was obtained at the University Metropolitana of Barranquilla prior to the data collection.

## Results

### Characteristics of study population

The present study enrolled 55 participants affected with leprosy (35 participants) or leishmaniasis (20 participants), of whom 56% were men and 44% women. One of the participants left due to personal reasons. She only participated in the SRQ, not in the other questionnaires and the group discussions. The average age of participants was 51 (range 19–85) and the participants had an average of 8 years of education (ranging from 0 to 20 years) (Table 1).

**Table 1:** Summary of participant characteristics.

Leishmaniasis	20	Sex	15 male/ 5 female
		Age (years)	19–63 (average 44)
		Location of health program	Cúcuta
		Education (years)	8–20 (average 10)
Leprosy	35	Sex	16 male/ 19 female
		Age (years)	28–85 (average 54)
		Location of health program	Cartagena 24/ Cúcuta11
		Education (years)	0–16 (average 7)

A minority of the participants were married at the time of the interview (29%). Others were never married (25%) or cohabiting (25%). Most participants were self-employed (33%), while others had paid work (20%) or worked in housekeeping (20%). Sixteen percent of all participants indicated to be unemployed due to health reasons, whom all were affected by leprosy. More information about the participants’ characterizations is provided in supporting data S1 Table.

### Results: Clinical profile

#### Conceptual equivalence

The questionnaire is straightforward about body structures and function (Table 2), there were no different interpretations of concepts given according to the interviewers and observers. It was clear to all participants that the domain of the questionnaire was about their body function and structure. During the interviews the participants shared their opinion about the relevance and importance of the questionnaire to themselves and others.

**Table 2:** Occurrence of comprehension difficulties and action needed of the clinical profile. (N = 54). A cut-off of 20% was used to determine if a question was well understood. Questions that exceed 20% cut-off are marked in red.

Question		Examples	Rephrasing	E&R	Total no. of patients with difficulties	%
1	Do you have any problem seeing things?	5	4	1	10	18.5%
2	Do you have any problem hearing sounds or voices?	1	0	0	1	1.9%
3	Do you have any problems with your skin? E.g. sensitivity or irritation	11	6	3	20	37.0%
4	Do you have any skin lesions?	10	0	1	11	20.4%
5	Do you have any open wounds?	0	0	0	0	0.0%
6	Do you experience pain in your chest and/or palpitations or are you easily tired?	5	4	0	9	16.7%
7	Do you have any problems breathing?	1	3	0	4	7.4%
8	Are you easily out of breath or do you have difficulty breathing?	0	1	0	1	1.9%
9	Do you have any problems swallowing food? E.g. choking or food that gets stuck?	1	0	1	2	3.7%
10	Do you have any problems with bowel movements or abnormal appearance of your stool? E.g. blood or worms	6	1	5	12	22.2%
11	Do you pass too little urine or is there blood in your urine, or do you have pain when you try to pass urine?	1	0	2	3	5.6%
12	Do you have tremors, unusual movements, epileptic fits or problems controlling movements?	1	0	2	3	5.6%
13	Do you often experience pain?	5	3	3	11	20.4%
14	Do you experience pain, loss of feeling or weakness in your arms or legs?	4	2	0	6	11.1%
15	Do you have any problems with insufficient strength in your arms or legs?	0	2	0	2	3.7%
16	Do you have any problems with movement of your arm, hand, wrist, elbows or shoulders?	3	1	2	6	11.1%
17	Do you have any problems with movement of your leg, foot or knees?	2	2	0	4	7.4%
18	Do you have an impairment (disability/limitation)?	3	3	4	10	18.5%
19	If yes, please describe	0	2	0	2	3.7%
20	If not, does your disease or condition cause limitations in your daily activities or restrictions in your contact with others?	2	3	1	6	11.1%

#### Item equivalence

During the interviews, the majority of the participants shared their opinion about the relevance of the questionnaire to their particular situation.

A participant said:

“The questions are important, because they study how a person stays after this (the disease)”

(male, 22)

Furthermore, the participants said the questions asked are acceptable to them and the general Colombian population. Also during the administration of the tool, no inconvenience was observed.

#### Semantic equivalence

For this questionnaire several problems were noted. First of all, the participants were not always able to answer the questions because some words can have multiple meanings, for example in question 1:

Q1. Do you have any problems seeing things?

The term “seeing things” is not clear and specific. This can be interpreted in multiple ways (literally/figuratively) and should be accompanied by examples. Secondly, some questions were too medical and not understandable for individuals with limited education. For example question 10:

Q10. Do you have any problems with bowel movements or abnormal appearance of your stool?

This question is sometimes hard to answer by the participants because they were not familiar with the term “bowel” or it might be unknown what situation should be considered as normal.

A few participants mentioned to have difficulties with question 3:

Q3. Do you have any problems with your skin? (for example sensibility or irritation)

It was unclear what was meant by “problems with your skin”. Furthermore, according to the researchers the term “sensibility” used in this Spanish version (“sensibilidad”) does occur in Colombia, but is perhaps not common for the target population. To get the questions more clear to the participants, one participant said:

“*The question about sensibility*, *people might not understand that term*. *To be more clear*, *change the word or at least explain that it is about feeling*.”

(*female*, *54*)

Overall the opinion was that a few questions of this questionnaire should be more specific. One participant for example said:

“*The questions are clear*, *but needs to be more specific*. *For example*, *the question about pain in my legs was not clear*, *because I have pain in my legs but that is because of the veins*.”

(*female*, *58*)

Question 12 was too difficult because not all participants knew what epilepsy is. The native speaking interviewer said:

“*I think the terminology used might have been too specific and quite punctual for the participants to answer some questions*. *Since some participants*, *seeing their education*, *do not know how to interpret the question*, *they answer yes or no without understanding them*.”

(*Jose*, *interviewer*)

This was his observation based on the body language and facial expressions during the assessment of the tool. In relation to this a participant said:

“*I am from the countryside*. *As you know*, *people from the countryside are more shy*. *If there is a question they don’t understand this might be a problem*.”

(*female*, *45*)

A participant mentioned:

“*It depends on the person’s intellectual ability*. *For people without education this might be difficult*. *There are people who don’t see*, *hear or read about these things*.”

(*male*, *49*)

#### Operational equivalence

The Clinical Profile was administered to all participants except for one (n = 54), with an average administration time of 7.4 minutes (ranging from 2 to 24 minutes). According to the participants this was an acceptable time for this tool.

The format of the Clinical Profile is very straightforward. During the interview most participants mentioned the response scales were perceived as helpful to answer the questions. Only one participant mentioned to have difficulties with the response scales. The answer possibilities were not terms he would use in general life, and he suggested using scales from 1 to 3, light, moderate and severe, respectively.

During the interview several of the participants noted that the questions were clearly formulated. However, the questionnaire is unable to distinguish between symptoms caused by the NTD or other causes. For example:

“*The formulation of the questions was clear*, *but it is not clear if the question is about before*, *during or after the disease*. *For example*, *I answered yes to the question if I have problems with my eyes*, *but that is unrelated to my disease*”.

(*female*, *54*)

The participant advised to give more instructions about the causes and time before starting the questionnaire.

Overall, the participants had some difficulties with the Clinical Profile. Of the 20 questions, only question 5 was fully understood by all 54 participants, while 4 questions were not understood by at least 20% of the participants (see Table 2). The questions shown in red need additional attention.

### Results: SRQ

#### Conceptual equivalence

This study did not assess the conceptual equivalence of the SRQ itself since the SRQ-20 had previously been used successfully in Colombia [32, 54–56]. Therefore, mental health has been established as a concept in Colombia. The participants said the concepts assessed with this questionnaire are relevant and important for their situation.

#### Item equivalence

The participants had a positive impression about the SRQ. A participant said the following about the relevance of the questionnaire and the items used:

“*The questionnaire is very important because it identifies problems we have*. *It is important because it points out how we handle the situation*”.

(*male*, *63*)

Another participant said the questions were easy for him to answer:

“Easy, because suddenly the questions gave me the possibility to truly say what I feel or not feel.”

(male, 60)

This questionnaire contains sensitive items, however, the interviewers did not observe frequent discomfort of the participants while answering these questions. According to the participants themselves, the items were perceived as acceptable and they were able to relate to the questions asked.

#### Semantic equivalence

The questions were understood directly by almost all participants and did not need additional examples or rephrasing (see Table 3). The participants said the level of Spanish was understandable for the general population of Colombia. Some participants needed examples or rephrasing for question 7 and 8, however the majority of the participants did not.

Q7. Is your digestion poor?
Q8. Do you have trouble thinking clearly?

**Table 3:** Occurrence of comprehension difficulties and action needed of the SRQ. (N = 55) A cut-off of 20% was used to determine if a question was well understood. All questionnaire items were well understood according to less than 20% of participants encountering any comprehension difficulties.

Question		Examples	Rephrasing	E&R	Total no. of patients with difficulties	%
1	Do you often have headaches?	0	0	0	0	0.0%
2	Is your appetite poor?	0	0	0	0	0.0%
3	Do you sleep badly?	0	0	1	1	1.9%
4	Are you easily frightened?	0	1	0	1	1.9%
5	Do your hands shake?	0	0	0	0	0.0%
6	Do you feel nervous, tense or worried?	0	0	0	0	0.0%
7	Is your digestion poor?	1	4	0	5	9.3%
8	Do you have trouble thinking clearly?	1	3	2	6	11.1%
9	Do you feel unhappy?	1	0	0	1	1.9%
10	Do you cry more than usual?	0	0	0	0	0.0%
11	Do you find it difficult to enjoy your daily activities?	1	1	2	4	7.4%
12	Do you find it difficult to make decisions?	1	0	1	2	3.7%
13	Is your daily work suffering?	1	1	1	3	5.6%
14	Are you unable to play a useful part in life?	0	2	3	5	9.3%
15	Have you lost interest in things?	0	1	2	3	5.6%
16	Do you feel that you are a worthless person?	0	0	0	0	0.0%
17	Has the thought of ending your life been on your mind?	0	1	0	1	1.9%
18	Do you feel tired all the time?	0	1	0	1	1.9%
19	Do you have uncomfortable feelings in your stomach?	0	2	0	2	3.7%
20	Are you easily tired?	0	0	1	1	1.9%

#### Operational equivalence

The participants found it easy to answer yes/no to all 20 questions. Although only yes/no answers were required, the participants were sometimes more talkative and shortly explained their answers in a few words. With regard to the instructions, the interviewer noticed a difficulty with several participants. Although the interviewer explained at the beginning of the interview that the questions should be related to their health, their answers sometimes indicated that they did not relate them to their health situation and needed to be reminded to this.

### Results: WHOQOL-BREF

#### Conceptual equivalence

The WHOQOL-BREF is part of the domain of personal factors of the ICF and covers physical health, psychological health, social relationships and environment. The researchers’ opinion was that these concepts of quality of life were as relevant and important to the target culture as it is to the source culture, because the answers of the participants given during administration of the tool showed the participants were clearly affected regarding these concepts in their quality of life caused by the NTD. Furthermore, the WHOQOL-BREF has been used before in Colombia, which indicates that the concepts of this questionnaire are present in this target culture [57].

When the interviewer asked why the participant thought this questionnaire is important, the following response of a participant was shared by most participants:

“*Very interesting questions*, *covering all aspects of the person*, *thus very complete*”

(*female*, *41*)

#### Item equivalence

During the interviews the majority of the participants shared their opinion about the relevance of the questionnaire and its items to their particular situation. One participant mentioned:

“*It is very important these questions are asked*, *because in general the doctor only asks for the clinical effects*. *The effects of the disease goes further than only clinical*, *it has a great influence in the quality of life for patients like me*.”

(*female*, *41*)

However, more than half of the participants had difficulties with question 14:

Q14. To what extent do you have the opportunity for leisure activities?

The researchers think this is due to the Spanish word used for leisure: *actividades de ocio*, which could be a semantic issue. However, this could also be because many people in Colombia are not accustomed to having leisure activities unlike what is seen in high-income countries and therefore be an item equivalence issue.

One of the participants said to be uncomfortable with speaking about the aspects addressed in this particular questionnaire concerning his disease in general. However, he recognized the importance of these questions and was glad to help with this study.

#### Semantic equivalence

According to the researchers, the transfer of meaning across languages was not completely satisfactory. For example, the researcher noticed several participants who had difficulties with question 13:

Q13. How available is the information you need in your day-to-day life?

According to the researchers this is because it is unclear for the participants what is meant by “information you need”. This question needed many examples in order to understand the question. Rephrasing this question in a way it is directly clear what is meant, or giving some examples supporting this question would be a solution. Question 14, already mentioned in the section on item equivalence, is also an issue for the semantic equivalence. This is because the word used for leisure, *actividades de ocio*, was not directly understood by the participants. A possible solution can be to accompany this question with examples or change the word *ocio* into *recreo* for example. Another important question concerned the semantic equivalence of question 16:

Q16. How satisfied are you with your sleep?

The Spanish word used in the translation of this questionnaire is “*sueño*”. This word can have several meanings in the Spanish language and is often conceived of as dreaming instead of sleeping. The interviewer often explained this question by replacing this term by “*dormir*", which is directly understood by the participant. Replacing this word in the questionnaire would possibly solve this problem.

Question 17 was not clear to approximately 20% of the participants. This was probably due to the question being too vague. Examples and rephrasing were necessary, probably because the terms ability and daily living activities were not specific enough.

Q17. How satisfied are you with your ability to perform your daily living activities?

According to the interviewer, the level of Spanish generally used in this questionnaire was too difficult to understand by the study population in Colombia. This leads to wrong answers given to the questions. In the cognitive interview after applying the questionnaire, most participants gave short answers, possibly to avoid giving the wrong answers, because they did not always understand the questions. Most participants said not to have problems with the questions, however, the results of the observations made during the interview with the tool indicated differently (Table 4).

**Table 4:** Occurrence of comprehension difficulties and action needed of the WHOQOL-BREF. (N = 54). A cut-off of 20% was used to determine if a question was well understood. Questions exceeding the 20% cut-off are marked in red.

Question		Examples	Rephrasing	E&R	Total no. of patients with difficulties	%
1	How would you rate your quality of life?	1	2	2	5	9.3%
2	How satisfied are you with your health?	0	1	2	3	5.6%
3	To what extent do you feel that (physical) pain prevents you from doing what you need to do?	2	4	3	9	16.7%
4	How much do you need any medical treatment to function in your daily life?	9	2	4	15	27.8%
5	How much do you enjoy life?	0	0	0	0	0.0%
6	To what extent do you feel your life to be meaningful?	0	4	1	5	9.3%
7	How well are you able to concentrate?	4	4	2	10	18.5%
8	How safe do you feel in your daily life?	0	2	7	9	16.7%
9	How healthy is your physical environment?	7	4	4	15	27.8%
10	Do you have enough energy for everyday life?	3	1	0	4	7.4%
11	Are you able to accept your bodily appearance?	1	1	1	3	5.6%
12	Have you enough money to meet your needs?	2	3	1	6	11.1%
13	How available to you is the information that you need in your day-to-day life?	7	4	12	23	42.6%
14	To what extent do you have the opportunity for leisure activities?	11	4	15	30	55.6%
15	How well are you able to get around?	1	6	1	8	14.8%
16	How satisfied are you with your sleep?	3	4	2	9	16.7%
17	How satisfied are you with your ability to perform your daily living activities?	5	7	0	12	22.2%
18	How satisfied are you with your capacity for work?	4	3	1	8	14.8%
19	How satisfied are you with yourself?	0	2	2	4	7.4%
20	How satisfied are you with your personal relationships?	3	3	1	7	13.0%
21	How satisfied are you with your sex life?	0	1	0	1	1.9%
22	How satisfied are you with the support you get from your friends?	0	1	0	1	1.9%
23	How satisfied are you with the conditions of your living place?	3	4	3	10	18.5%
24	How satisfied are you with your access to health services?	5	4	0	9	16.7%
25	How satisfied are you with your transport?	1	2	2	5	9.3%
26	How often do you have negative feelings such as blue mood, despair, anxiety, depression?	3	1	2	6	11.1%

#### Operational equivalence

The WHOQOL-BREF has been administered to all participants except for one, with an average administration time of 9.8 min (ranging from 5 to 15 min). This administration time was perceived as acceptable by the majority of the participants. However, a few participants mentioned this was long in combination with other instruments from the toolkit.

Several difficulties were found concerning the response options. A significant number of participants used different words to answer the question than provided by the questionnaire. Their answers were diverse and the respondents needed to be reminded of the answer options repeatedly. Interestingly, during the cognitive interview after the questionnaire, participants did not mention having problems with these response options used in the questionnaire. Only a minority of the participants pointed out that it was confusing that the response scales changed during the questionnaire (four different scales, changing 5 times in the WHOQOL-BREF).

Overall, this questionnaire was not easily understood by a majority of the participants. Of the 26 questions, only question 5 was fully understood by all 54 participants, while 7 questions were not understood by at least 10 participants (Table 4).

### Results: WHOQOL-DIS

#### Conceptual equivalence

The WHOQOL-DIS was administered to 15 participants. This questionnaire was only administered when question 18 of the Clinical Profile, “Do you have an impairment (disability/limitation)?” was answered with “yes”. Although to our knowledge the WHOQOL-DIS has not been used in Colombia before, previous research shows that the concepts assessed with this questionnaire are present in the Colombian culture [58, 59].

Not all participants agreed that this questionnaire was important for their particular situation; however, they can imagine this questionnaire is important for other people affected by NTDs. In these cases the participants mentioned their situation was not severe enough for the questionnaire to be relevant for them.

#### Item equivalence

The items used in this questionnaire seemed relevant to the target culture, because the participants were able to relate to the questions asked. For example, several participants answered to question 37 that they were not satisfied with the possibilities to participate in social activities due to their NTD. In addition, several patients also mentioned to encounter negative effects on finding opportunities to work because of the NTD (question 40).

The WHO stated in 1997:

“*Quality of life is defined as individual’s perceptions of their position in life in the context of the culture and value systems in which they live and in relation to their goals*, *expectations*, *standards and concerns*”

(*WHO*, *1997*)

The participants indicated they were affected in their quality of life by the disabilities due to the NTD in relation to their goals and expectations, showing these items are relevant to them. For example, several participants replied to question 39 that they were not satisfied in relation to their goals and expectations:

Q39. Do you feel that your dreams, hopes and wishes will happen? *For example*, *do you feel you will get the chance to do the things you want*, *or get the things you wish for*, *in your life*?

Most participants shared their opinion about the acceptability of the items. During the cognitive interview, the interviewer asked the participants if there was a word or a question that made them feel uncomfortable. Like one woman of 56 affected by leishmaniasis, most participant answered this question with “*No*, *none*”.

#### Semantic equivalence

The meaning of the questions was not directly clear to many participants and additional examples or rephrasing were needed (Table 5). The most frequent comment of the participants during the interviews was that the questions are too broad. For example:

Q27. Does your disability have a negative (bad) effect on your day-to-day life?

**Table 5:** Occurrence of comprehension difficulties and action needed of the WHOQOL-DIS. (N = 15). A cut-off of 20% was used to determine if a question was well understood. Questions that exceed the 20% cut-off are marked in red.

Question		Examples	Rephrasing	E&R	Total no. of patients with difficulties	%
27	Does your disability have a negative (bad) effect on your day-to-day life?	3	1	3	7	46.7%
28	Do you feel that some people treat you unfairly?	0	1	1	2	13.3%
29	Do you need someone to stand up for you when you have problems?	2	3	0	5	33.3%
30	Do you worry about what might happen to you in the future? For example, thinking about not being able to look after yourself, or being a burden to others in the future.	0	0	0	0	0.0%
31	Do you feel in control of your life? For example, do you feel in charge of your life?	0	1	0	1	6.7%
32	Do you make your own choices about your day-to-day life? For example, where to go, what to do, what to eat.	0	1	0	1	6.7%
33	Do you get to make the big decisions in your life? For example, like deciding where to live, or who to live with, how to spend your money.	0	0	1	1	6.7%
34	Are you satisfied with your ability to communicate with other people? For example, how you say things or get your point across, the way you understand others, by words or signs.	0	1	1	2	13.3%
35	Do you feel that other people accept you?	0	0	0	0	0.0%
36	Do you feel that other people respect you? For example, do you feel that others value you as a person, and listen to what you have to say?	1	1	0	2	13.3%
37	Are you satisfied with your chances to be involved in social activities? For example, meeting friends, going out for a meal, going to a party etc.	0	3	1	4	26.7%
38	Are you satisfied with your chances to be involved in local activities? For example, being part of what is happening in your local area or neighbourhood.	3	1	0	4	26.7%
39	Do you feel that your dreams, hopes and wishes will happen? For example, do you feel you will get the chance to do the things you want, or get the things you wish for, in your life?	0	0	1	1	6.7%

Here it was not clear which activities were meant by day-to-day life. Additional examples helped the participants to understand the question. Advice of the interviewer for this questionnaire is to include a few examples.

#### Operational equivalence

The average administration time was 8.9 min (ranging from 6 to 19 min). The participants with a relatively short administration time were satisfied with this administration time. The participants with a long administration time considered this to be too long.

The response scales used were not helpful to the participants and did not correspond to the answers the participants gave. First, the words used as answer options are known to the participants, however, they are not the first things that come to mind. Second, it was confusing for the participants that the response scales changed during the administration of the questionnaire. Furthermore, one of the interviewers said:

“*The answer options given were not clear*. *If a situation was seen as ‘normal’*, *some patients would answer this as medium*. *However*, *other patients would answer excellent if the situation was seen as normal*.”

(*Jose*, *interviewer*)

### Results: The interview as a whole

Most participants mentioned during the group discussions that the tools of the toolkit are very important for their particular situation, because these are important topics related to their health condition that generally no person asks about. The data generated would be very useful to improve their daily life with disabilities due to NTDs. The average administration time was 7.0 min per questionnaire (range between 1.4 to 24 min). Overall, the participants had the impression the toolkit could be a relevant tool for their situation. A participant answered regarding the relevance for their particular situation:

“*Yes*, *what I tell you is to help the study and to improve the healthcare service to be excellent*”.

(*male*, *54*)

## Discussion

The aim of this study was to culturally validate the Clinical Profile, SRQ, WHOQOL-BREF & WHOQOL-DIS in Colombia among people affected by NTDs, in order to include these health-related questionnaires as tools in the cross-NTD toolkit. The partial validation with the use of the qualitative equivalences, was based on the framework for cultural validation of Herdman *et al*., using conceptual-, item-, semantic- and operational equivalence. The main findings of these equivalences are presented in Table 6.

**Table 6:** Main findings of the equivalences per questionnaire.

	Equivalences			
Tools	Conceptual	Item	Semantic	Operational
Clinical Profile	The concepts are present, relevant and important	The items are not influenced by culture	Some difficulties: words can have multiple meanings or too medical. However, the majority of items was well understood.	Format is straightforward and answer options seen as useful
SRQ	The concepts are present, relevant and important	The items are acceptable even though these might be sensitive. Participants can relate to the items	The language and meaning were understood directly by almost all participants	Easy to use yes/no questions
WHOQOL-BREF	The concepts are present, relevant and important	The items were seen as relevant for their particular situation.	Not as desired, considerable examples and rephrasing were needed	Responses of participants differed to the provided options. Also difficulties with multiple changes in response options during the questionnaire
WHOQOL-DIS	Although this questionnaire was not seen as important by some participants for themselves, they do think this is relevant for others affected by NTDs.	Items are perceived as acceptable and most participants could relate to the items.	The meaning of questions was not directly clear	The response scales were not seen as helpful and the administration time sometimes too long.

### The validity of the clinical profile

This study has shown that the Clinical Profile is a useful instrument to measure the domain of body function and structure. Many measurement methods to assess the clinical features of leprosy and leishmaniasis are only able to be administered by physicians [60, 61]. This questionnaire can be used by non-physicians and allows participants to provide their own answers. The conceptual and item equivalences were reached. This study showed that the concepts are present, relevant and important in the cultural settings of Colombia, indicating the used construct of the questionnaire is likely to be equally valid in both cultures. The items contain subjects that are not influenced by culture and can therefore be considered as equivalent.

For this questionnaire there were a few problems concerning the semantic equivalence. The participants were not always able to answer the questions because some words can have multiple meanings. In other cases, the level of Spanish might have been too medical. This was not noticed in the pilot testing, which might be due to varying levels of education. This would also explain why participants did not always know the answer to a question, for example to question 18, asking whether the participant has impairments. Assessment of the operational equivalence focuses on the format of the questionnaire, mode of administration and measurement methods. The format of the Clinical Profile is very straightforward. The participants perceived the answer options as shown in the questionnaire (S1 and S2 Appendices) as helpful.

The questionnaire was developed in 2015 by Van ‘t Noordende and colleagues, and had only been validated in Brazil. The results of their validation study support our finding of question 18 being too difficult to answer by several participants [18]. As mentioned by Van ‘t Noordende and colleagues, a limitation of this questionnaire is that it does not differentiate between clinical symptoms caused by the NTD or by other factors, such as co-morbidity of aging. The participants of this study also mentioned this. However, from the perspective of the ICF, this is not a limitation. When viewing a person’s condition/disability holistically, the cause of the particular problem does not matter.

The partial validity of the questionnaire in its current state can be considered acceptable, provided the language is simplified, and that examples or criteria for “normal” are added. The fact that the toolkit is used as a face-to-face interview with the participants is an advantage to elucidate questions that are not directly understood by the participants. However, the need for explanation or examples should be minimized. Therefore, for the use of the Clinical Profile in Colombia, it is recommended to change at least 4 of the questions in order for the participant to understand them as they were meant. Suggestions for adaptions are given in S2 Table.

### Validity of the SRQ

The SRQ was developed to screen for mental disorders [24]. Based on the results of the present study, the SRQ has good partial validity for the equivalence that were evaluated to use with persons affected by leishmaniasis and leprosy in Colombia. Based on the interviews and the evaluation with the researchers in a group discussion, we conclude that the conceptual and item equivalences for this questionnaire are reached. We concluded also that the level of Spanish is suitable for the study population since the questions were understood directly by almost all participants. Operational equivalence was reached as well. The participants found the yes/no answer options easy to use for all 20 questions.

The validation study in Brazil supports the findings that the SRQ is easily administered to several levels of economic classes in society, because of the yes/no answer options. Van ‘t Noordende and colleagues noted that the topic on suicide used in the questionnaire is often taboo in developing countries and that this might have been the reason in their study that no participants answered ‘yes’ to this question. This is in contrast to the present study, where a minority of participants did indicate having had thoughts of committing suicide in the past. However, there was no inconvenience observed while asking these questions. The SRQ has previous been validated in Colombia in primary health care, indigenous Colombians and young urban Colombians [24, 32–34]. They support our findings that the SRQ is an easy to use questionnaire that can be used for routine screenings. Moreover, the tool has been validated in low and middle-income countries, such as in South Africa in 2015 [62]. This study assessed a sample of 200 patients of emergency centres in Cape Town, South Africa. They measured the psychometric properties, which are not measured in the present study as part of the measurement equivalence [62]. Their results show a good internal consistency (0.84), moderate sensitivity (63%) and good specificity (88%) with a cut-off score 7/8 [23, 62]. Also other studies, including a systemic review, consider the SRQ to be reliable, accurate and consistent [62–65]. The results of the present study show the SRQ has good partial validity for the equivalence that were evaluated among persons affected by leprosy and leishmaniasis in the cultural setting of Colombia. This is not surprising, because this questionnaire was first validated in four different developing countries, one of them being Colombia [24]. However, they only provide the global results and the performance in Colombia was not presented. The current study validated the response scale among participants affected with an NTD. The findings show that this instrument is suitable to be included in the toolkit without any rephrasing.

### Validity of the WHOQOL-BREF

The WHOQOL-BREF has been used in multiple studies in Colombia [40–43]. The conceptual and item equivalences are reached, based on the fact that these concepts and the same questionnaire were studied before, the opinions of the interviewers and the group discussions with the participants. This is supported by the findings of a study assessing the importance of the items in the WHOQOL-BREF, among 4804 respondents, conducted in 14 developing and developed countries and using 12 languages including Spanish [39]. However, the semantic equivalence was not found to be sufficient in the present study among people affected by leprosy and leishmaniasis. The results show that many participants did not understand several questions without additional examples or rephrasing. Specifically question 14 was not understood by more than half (56%) of the participants. This might be due to the use of the word ‘leisure’, which should be replaced with a suitable synonym, more understandable for the target group. The inadequate semantic equivalence is in contrast to the findings of a validation study performed in Chile, another Spanish speaking country of South America [66]. They did not have problems with the semantic equivalence and concluded that the WHOQOL-BREF has acceptable validity. Also, a validation study of the Spanish WHOQOL-BREF conducted in multiple Spanish speaking countries and different health-conditions concluded this version to be suitable [67, 68]. This difference might be explained by the differences in the level of education of the respondents and the different use of words in the Spanish language, emphasizing the importance of cultural validation. Suggestions for changing questions that were not directly understood by at least 20% of the participants are provided in S4 Appendix. The operational equivalence of the WHOQOL-BREF was assessed during the interview. Interestingly, a significant number of participants did not use the options in the response scales provided by the questionnaire. Often other answers were given. Furthermore, the fact that the response options changed during the questionnaire was experienced as confusing. A solution would be to introduce flashcards placed in front of the respondents, which can be changed during the interview to attract their attention.

The pilot study performed in Brazil also showed many participants had difficulties understanding the questions of this tool [18]. They recommended that the WHOQOL-BREF translation should be validated with representative sample of people with limited education, as has been done in this study, although this was not a selection criteria.

Overall, the participants found the total administration time acceptable, although some thought it was slightly too long. The WHOQOL-BREF in its current state is not directly suitable to be used with the target group in Colombia without rephrasing a few questions. However, the participants noted the questionnaire was important for their particular situation. Therefore, it is recommended to rephrase the concerned questions in the questionnaire (S3 Table), design flash cards with answer options and then re-test the relevant aspects of this tool.

### Validity of the WHOQOL-DIS

To our knowledge, the WHOQOL-DIS has not yet been applied before among Colombian people. The conceptual and item equivalence in this study were satisfactory. Although this particular questionnaire has not been used in Colombia before, several studies have been conducted to measure the quality of life of people living with a disability by using other tools [59, 69, 70]. During the interviews in this study most participants answered “yes” to a question asking whether they thought the questionnaire was relevant and important. The items used to measure the quality of life living with a disability, were perceived as acceptable by all participants. The semantic equivalence is not yet as desired. The meaning of the questions was not immediately clear to many participants and additional examples or rephrasing were needed. This is in contrast to the findings of the previous study performed in other Spanish speaking countries [44]. Their results show that there were fewer difficulties with understanding the questions, however; this study was conducted in Spain. This shows that although a tool can exist in a Spanish version, cultural validation is still very important. The pilot testing of the questionnaire did not predict the semantic problems found. This might be explained by the differences in educational background of the participants in the pilot and in the subsequent study. However, this questionnaire was only applied to 15 participants in the present study and this might not be an adequate representation of the target group. Further research is needed to come to a conclusion about validity of this module for the cultural settings of Colombia.

The pilot study in Brazil concluded the questionnaire to be suitable for the NTD toolkit. However, they recommended a visual flashcard for the answering options, because the options used to answer changed during the questionnaire. As we recommended this for the WHOQOL-BREF, this would be a good addition for this tool as well.

The WHOQOL-DIS was not yet found suitable to be included in the toolkit. Adapting and retesting of this questionnaire is highly recommended to gain more knowledge about how to adjust this questionnaire to the Colombian context (S4 Table). Depending on the severity of the participants’ condition due to the NTD, this questionnaire will be an interesting tool to include in the toolkit, because this covers a domain not covered by the other tools.

A variety of studies have recognized that, to reach equivalence between the original questionnaire and the target version, instruments used across cultures should not only be translated in a linguistically correct manner. Rather, they should additionally be adapted culturally to maintain the content validity of the instruments at the conceptual level amongst various cultures. This may require changes in actual wording in order to preserve meaning across cultures. Different standard examples may also be needed or the use of answer aids, such as flash cards with pictorial answer options. However, in case any major changes are proposed these should be tested also elsewhere in order to retain equivalence of results.

### Limitations of the current study

During the interviews on the 3^rd^ day in Cartagena, some of the participants were able to hear each other speak, which may have influenced the data, because of the sometimes sensitive questions. Furthermore, on this day, several patients have been waiting longer than planned. This might have influenced the data because some of the participants became impatient and may have given answers even if they did not understand the questions in order to finish the interview fast. One participant left during the interview because of time. This data is not included in the study.

Importantly, adding non-standard examples to clarify a question for some patients but not all, may have introduced a measurement bias. This applies for all questionnaires. It is therefore important to use questionnaires that are applicable to and understandable for the whole target group to generate comparable data. This may require inclusion of certain standard examples that are used for all.

While the number of participants was small (n = 15), this number was sufficient for the qualitative validation. Q18 may not have been understood directly first time round, but with additional examples or rephrasing, respondents still understood the question. So the answer ‘Yes’ can still be considered valid. For the future use of the Clinical Profile, such examples should be added as standard, but for the purpose of selecting persons with a disability, we considered the question adequate as long as it was understood with additional explanation or examples.

NTDs have several aspects in common: geographical distribution, prevention and treatment, stigma, disability and rehabilitation of people living with the consequences of NTDs. This study in Colombia has limited generalizability to other countries with different cultures. However, this study is one in a series in which the different instruments are tested in different cultures and languages and among persons with different NTDs. Together these studies will provide the necessary evidence base for cross-NTD and cross-cultural validity.

## Conclusion

Cultural validation is necessary for health-related questionnaires when the purpose is to use these tools in a different cultural setting or with a different target group than the one the instrument was developed in or for. In the present study, the validity of the tools was assessed for the cultural settings of people affected by NTDs in Cartagena and Cúcuta, Colombia. We conclude that with regard to the four equivalences evaluated, the SRQ is valid and can be used in the toolkit without any changes for the cultural settings of Colombia. The Clinical Profile is perceived as a useful tool, but needs to be retested with the suggested adaptations. The WHOQOL-BREF and WHOQOL-DIS need additional adaptations as well. However, because these instruments are designed by the WHO, only minor adaptations can be made in these questionnaires. After the recommended changes in the Clinical Profile, WHOQOL-BREF and WHOQOL-DIS have been made, these questionnaires need to be retested to confirm their semantic, item and operational equivalences for the use in Colombia. We also recommend to assess measurement equivalence in future quantitative studies to complement the results provided by this research.

Spanish is a language spoken in many countries in the world, mainly in Latin America. Because of subtle differences in the dialect used in different areas, we recommend to pilot the translation of each instrument before use in new area in Colombia, as well as in other Spanish-speaking countries. In future studies it would also be interesting to compare participants affected by NTDs to healthy controls in the same population.

## Supporting information

S1 Table Participants’ characteristics. (DOCX)

S2 Table Clinical profile suggested changes. (PDF)

S3 Table WHOQOL-BREF suggested changes. (PDF)

S4 Table WHOQOL-DIS suggested changes. (PDF)

S1 Appendix Clinical profile Spanish. (PDF)

S2 Appendix Clinical profile English. (PDF)

S3 Appendix SRQ Spanish. (PDF)

S4 Appendix SRQ English. (PDF)

S5 Appendix WHOQOL-BREF & WHOQOL-DIS Spanish. (PDF)

S6 Appendix WHOQOL-BREF & WHOQOL-DIS English. (PDF)

S7 Appendix Interview guide Spanish. (PDF)

S8 Appendix Interview guide English. (PDF)

S9 Appendix Focus group guide Spanish. (PDF)

S10 Appendix Focus group guide English. (PDF)

S11 Appendix Informed consent. (PDF)

S12 Appendix COREQ-checklist and research group. (PDF)
