# Supplementary material for: Validation of a cross-NTD toolkit for assessment of NTD-related morbidity and disability. A cross-cultural qualitative validation of study instruments in Colombia
Source: PLoS One. 2019 Dec 3;14(12):e0223042. doi: 10.1371/journal.pone.0223042 (PMC6890168; doi:10.1371/journal.pone.0223042)
Supplement: S1 Table — (DOCX) [file pone.0223042.s001.docx]

**S1 Table. Participants’ characteristics.**

| **Patiënt nr.** | **Health condition** | **Sex** | **Age (years)** | **Location of health program** | **Education (years)** | **Occupation** | **Marital status** |
| --- | --- | --- | --- | --- | --- | --- | --- |
| 1 | Lepra | male | 81 | Cartagena | 0 | unemployed due to healh conditions | widowed |
| 2 | Lepra | male | 73 | Cartagena | 0 | unemployed due to healh conditions | separated |
| 3 | Lepra | male | 70 | Cartagena | 5 | self-employed | currently married |
| 4 | Lepra | female | 53 | Cartagena | 5 | self-employed | cohabiting |
| 5 | Lepra | male | 61 | Cartagena | 7 | self-employed | separated |
| 6 | Lepra | female | 54 | Cartagena | 5 | house keeping | currently married |
| 7 | Lepra | male | 48 | Cartagena | 7 | self-employed | cohabiting |
| 8 | Lepra | male | 81 | Cartagena | 0 | other | cohabiting |
| 9 | Lepra | male | 38 | Cartagena | 7 | self-employed | separated |
| 10 | Lepra | male | 44 | Cartagena | 7 | self-employed | currently married |
| 11 | Lepra | female | 43 | Cartagena | 10 | self-employed | never married |
| 12 | Lepra | male | 29 | Cartagena | 15 | student | never married |
| 13 | Lepra | male | 64 | Cartagena | 5 | unemployed due to healh conditions | currently married |
| 14 | Lepra | male | 53 | Cartagena | 8 | self-employed | cohabiting |
| 15 | Lepra | male | 31 | Cartagena | 11 | unemployed due to healh conditions | currently married |
| 16 | Lepra | male | 79 | Cartagena | 0 | unemployed due to healh conditions | cohabiting |
| 17 | Lepra | male | 39 | Cartagena | 14 | paid work | separated |
| 18 | Lepra | female | 70 | Cartagena | 5 | house keeping | cohabiting |
| 19 | Lepra | female | 75 | Cartagena | 2 | house keeping | currently married |
| 20 | Lepra | male | 85 | Cartagena | 0 | self-employed | currently married |
| 21 | Lepra | male | 28 | Cartagena | 7 | unemployed due to healh conditions | never married |
| 22 | Lepra | male | 41 | Cartagena | 8 | other | never married |
| 23 | Lepra | male | 42 | Cartagena | 7 | unemployed due to healh conditions | separated |
| 24 | Lepra | male | 38 | Cartagena | 5 | self-employed | cohabiting |
| 25 | Leishmaniasis | female | 22 | Cucuta | 16 | paid work | separated |
| 26 | Lepra | female | 38 | Cucuta | 15 | house keeping | currently married |
| 27 | Leishmaniasis | female | 56 | Cucuta | 4 | self-employed | never married |
| 28 | Leishmaniasis | female | 53 | Cucuta | 2 | paid work | currently married |
| 29 | Leishmaniasis | female | 26 | Cucuta | 11 | house keeping | currently married |
| 30 | Leishmaniasis | female | 43 | Cucuta | 16 | paid work | never married |
| 31 | Leishmaniasis | male | 48 | Cucuta | 11 | unemployed due to healh conditions | never married |
| 32 | Lepra | male | 51 | Cucuta | 13 | self-employed | never married |
| 33 | Lepra | female | 47 | Cucuta | 10 | self-employed | widowed |
| 34 | Leishmaniasis | male | 24 | Cucuta | 12 | unemployed due to healh conditions | currently married |
| 35 | Leishmaniasis | male | 45 | Cucuta | 6 | paid work | separated |
| 36 | Leishmaniasis | male | 35 | Cucuta | 10 | paid work | never married |
| 37 | Leishmaniasis | female | 55 | Cucuta | 5 | house keeping | cohabiting |
| 38 | Leishmaniasis | male | 48 | Cucuta | 12 | self-employed | currently married |
| 39 | Lepra | female | 53 | Cucuta | 4 | house keeping | cohabiting |
| 40 | Leishmaniasis | female | 56 | Cucuta | 5 | paid work | currently married |
| 41 | Lepra | male | 46 | Cucuta | 13 | self-employed | never married |
| 42 | Lepra | male | 49 | Cucuta | 16 | paid work | never married |
| 43 | Leishmaniasis | female | 25 | Cucuta | 10 | house keeping | cohabiting |
| 44 | Leishmaniasis | male | 50 | Cucuta | 15 | unemployed due to other reasons | cohabiting |
| 45 | Lepra | female | 54 | Cucuta | 0 | house keeping | currently married |
| 46 | Lepra | female | 62 | Cucuta | 4 | paid work | cohabiting |
| 47 | Leishmaniasis | female | 50 | Cucuta | 8 | house keeping | cohabiting |
| 48 | Leishmaniasis | female | 56 | Cucuta | 8 | paid work | never married |
| 49 | Leishmaniasis | female | 63 | Cucuta | 6 | retired | currently married |
| 50 | Leishmaniasis | female | 54 | Cucuta | 20 | house keeping | never married |
| 51 | Lepra | female | 62 | Cucuta | 1 | self-employed | separated |
| 52 | Leishmaniasis | male | 19 | Cucuta | 15 | self-employed | currently married |
| 53 | Lepra | female | 64 | Cucuta | 5 | paid work | cohabiting |
| 54 | Leishmaniasis | male | 45 | Cucuta | 11 | student | never married |
| 55 | Lepra | male | 60 | Cucuta | 16 | self-employed | separated |
