# Supplementary material for: Validation of a cross-NTD toolkit for assessment of NTD-related morbidity and disability. A cross-cultural qualitative validation of study instruments in Colombia
Source: PLoS One. 2019 Dec 3;14(12):e0223042. doi: 10.1371/journal.pone.0223042 (PMC6890168; doi:10.1371/journal.pone.0223042)
Supplement: S2 Table — (PDF) [file pone.0223042.s002.pdf]

S2 Table. Clinical Profile suggested changes

Q nr	Original question	Suggested changes
Q1	¿ Tiene usted algún problema para ver las cosas?	Replace “las cosas” with “los objectos”
	Do you have any problem seeing things?	Replace “things” with “objects”
Q3	¿Tiene algunos problemas con su piel? Ej. sensibilidad o irritación	This question needed extra examples, specific examples about “sensibilidad” (sensitivity) “irritación” (irritation)
	Do you have any problems with your skin? E.g. sensitivity or irritation	Note: “sensibilidad” translated to English is both sensitivity and sensibility.
Q4	¿Tiene algunas lesiones en la piel?	Replace “lesiones” with “heridas”, or add an example
	Do you have any skin lesions?	Replace “lesions” with “wounds”
Q10	¿Tiene problemas intestinales? Movimientos o apariencia anormal de las heces? Ej. Sangre o parásitos	Add ”dolor en el estómago” as an example
	Do you have any problems with bowel movements or abnormal appearance of your stool? E.g. blood or worms	Add “pain in the stomach”
Q13	¿A menudo experimenta dolor?	
	Do you often experience pain?	Not specific, explain what kind of pain is meant, e.g. related to their NTD, which location or whether the pain is repetitive.
