# Supplementary material for: Validation of a cross-NTD toolkit for assessment of NTD-related morbidity and disability. A cross-cultural qualitative validation of study instruments in Colombia
Source: PLoS One. 2019 Dec 3;14(12):e0223042. doi: 10.1371/journal.pone.0223042 (PMC6890168; doi:10.1371/journal.pone.0223042)
Supplement: S3 Table — (PDF) [file pone.0223042.s003.pdf]

S3 Table. WHOQOL-BREF suggested changes

Q nr	Original question	Suggested changes
Q4	¿Usted necesita de algún tratamiento médico para desenvolverse en su vida diaria?	
	How much do you need any medical treatment to function in your daily life?	Provide examples about which treatment one should think about
Q9	¿Qué tan saludable es su entorno físico (clima, ruido, contaminación, atracciones)?	(Rephrase) ¿Tiene la idea de su entorno físico influye negativamente su salud? (clima, ruido, contaminación, atracciones)?
	How healthy is your physical environment?	(Rephrase) Do you have the idea of your physical environment negatively influencing your health?
Q13	¿Está disponible para usted la información que necesita en su día a día?	(Add example) Por ejemplo, cómo hacer frente a las complicaciones causadas por el NTD
	How available to you is the information that you need in your day-to-day life?	(Add example) For example, how to deal with the complications caused by the NTD
Q14	¿Hasta qué punto tiene usted la oportunidad de realizar actividades de ocio?	(Rephrase) ¿Su condición de salud influye negativamente en la posibilidad de participar en actividades recreativas?
	To what extent do you have the opportunity for leisure activities?	(Rephrase) Does your health condition negatively influence the possibility of participating in recreational activities?

Q17	¿Qué tan satisfecho (a) está con su capacidad de desempeño?	(Add to the sentence) "para realizar sus actividades diarias de vida"
	How satisfied are you with your ability to perform your daily living activities?	(Add to the sentence) "to perform your daily life activities"
