# Supplementary material for: Validation of a cross-NTD toolkit for assessment of NTD-related morbidity and disability. A cross-cultural qualitative validation of study instruments in Colombia
Source: PLoS One. 2019 Dec 3;14(12):e0223042. doi: 10.1371/journal.pone.0223042 (PMC6890168; doi:10.1371/journal.pone.0223042)
Supplement: S4 Table — (PDF) [file pone.0223042.s004.pdf]

S4 Table. WHOQOL-DIS suggested changes

Q nr	Original question	Suggested changes
Q27	¿Su discapacidad (discapacidad / limitación) tiene un efecto negative (malo) en su vida diaria?	
	Does your disability have a negative (bad) effect on your day-to-day life?	Add examples
Q29	¿Usted necesita que "alguien intervenga" cuando tiene problemas?	
	Do you need someone to stand up for you when you have problems?	Explain which type of problems by adding examples
Q37	¿Está usted satisfecho (a) con sus posibilidades de involucrarse en actividades sociales?<br><br>*Por ejemplo, para encontrar amigos, salir a comer fuera, ir a una fiesta, etc.*	
	Are you satisfied with your chances to be involved in social activities?<br><br>*For example, meeting friends, going out for a meal, going to a party etc.*	No suggestions
Q38	¿Está usted satisfecho (a) con sus posibilidades de participar en las actividades de su comunidad (local)? Por ejemplo, participar en lo que está sucediendo en su localidad o barrio.	
	Are you satisfied with your chances to be involved in local activities? For example, being part of what is happening in your local area or neighborhood.	Add examples for such activities
