# Supplementary material for: Validation of a cross-NTD toolkit for assessment of NTD-related morbidity and disability. A cross-cultural qualitative validation of study instruments in Colombia
Source: PLoS One. 2019 Dec 3;14(12):e0223042. doi: 10.1371/journal.pone.0223042 (PMC6890168; doi:10.1371/journal.pone.0223042)
Supplement: S1 Appendix — (PDF) [file pone.0223042.s005.pdf]

S1 Appendix. Clinical Profile Spanish

PERFIL CLÍNICO
**Juego de herramientas NTD - Funciones y estructuras corporales**
Identificación del participante: ___________ Diagnóstico: ____________ Perfil clínico: _______

ITEM	PREGUNTA	CATEGORÍA		EXTENSIÓN DEL DETERIORO (cuando "SÍ")	CÓDIGO ICF Y CATEGORÍA
1	¿Tiene usted algún problema para ver las cosas?	No<br>Si<br>No especificado<br>No aplica	0<br>1<br>99<br>88	( ) Leve<br>( ) Moderado<br>( ) Grave	b210 Visión
2	¿Tiene algún problema para escuchar sonidos o voces?	No<br>Si<br>No especificado<br>No aplica	0<br>1<br>99<br>88	( ) Leve<br>( ) Moderado<br>( ) Grave	b230 Audición
3	¿Tiene algunos problemas con su piel? Ej. sensibilidad o irritación	No<br>Si<br>No especificado<br>No aplica	0<br>1<br>99<br>88	( ) Leve<br>( ) Moderado<br>( ) Grave	b8. Funciones de la piel y estructuras relacionadas
4	¿Tiene algunas lesiones en la piel?	No<br>Si<br>No especificado<br>No aplica	0<br>1<br>99<br>88	( ) Leve<br>( ) Moderado<br>( ) Grave	s8. Estructuras de la piel y afines
5	¿Tiene alguna herida abierta?	No<br>Si<br>No especificado<br>No aplica	0<br>1<br>99<br>88	( ) Leve<br>( ) Moderado<br>( ) Grave	s8. Estructuras de la piel y afines
6	¿Siente dolor en el pecho y/o palpitaciones o se cansa fácilmente?	No<br>Si<br>No especificado<br>No aplica	0<br>1<br>99<br>88	( ) Leve<br>( ) Moderado<br>( ) Grave	b410 Corazón
7	¿Tiene algunos problemas respirando?	No<br>Si<br>No especificado<br>No aplica	0<br>1<br>99<br>88	( ) Leve<br>( ) Moderado<br>( ) Grave	b440 Respiración (respirar)
8	¿Se queda fácilmente sin respiración o tiene dificultad para respirar?	No<br>Si<br>No especificado<br>No aplica	0<br>1<br>99<br>88	( ) Leve<br>( ) Moderado<br>( ) Grave	s430 Sistema respiratorio
9	¿Tiene problemas para tragar los alimentos? Ej. Se ahoga o se atora con alimentos?	No<br>Si<br>No especificado<br>No aplica	0<br>1<br>99<br>88	( ) Leve<br>( ) Moderado<br>( ) Grave	b515 Digestión
10	¿Tiene problemas intestinales? Movimientos o apariencia anormal de las heces? Ej. Sangre	No<br>Si<br>No especificado	0<br>1<br>99	( ) Leve<br>( ) Moderado<br>( ) Grave	b525 Defecación

	o parásitos	No aplica	88		
11	¿Orina muy poco, hay sangre en la orina, o tiene dolor cuando trata de orinar?	No<br>Si<br>No especificado<br>No aplica	0<br>1<br>99<br>88	( ) Leve<br>( ) Moderado<br>( ) Grave	S610 Sistema Urinario
12	¿Tiene temblores, movimientos inusuales, ataques de epilepsia o problemas de control de movimientos?	No<br>Si<br>No especificado<br>No aplica	0<br>1<br>99<br>88	( ) Leve<br>( ) Moderado<br>( ) Grave	s110 cerebro
13	¿A menudo experimenta dolor?	No<br>Si<br>No especificado<br>No aplica	0<br>1<br>99<br>88	( ) Leve<br>( ) Moderado<br>( ) Grave	b280 Dolor
14	¿Siente dolor, pérdida de sensibilidad o debilidad en los brazos o las piernas?	No<br>Si<br>No especificado<br>No aplica	0<br>1<br>99<br>88	( ) Leve<br>( ) Moderado<br>( ) Grave	s120 Médula espinal y los nervios periféricos
15	¿Tiene disminución de fuerza en los brazos o las piernas?	No<br>Si<br>No especificado<br>No aplica	0<br>1<br>99<br>88	( ) Leve<br>( ) Moderado<br>( ) Grave	b730 Potencia muscular
16	¿Tiene algún problema con el movimiento de su brazo, mano, muñeca, codos o espalda?	No<br>Si<br>No especificado<br>No aplica	0<br>1<br>99<br>88	( ) Leve<br>( ) Moderado<br>( ) Grave	s730 extremidad superior (brazo, la mano)
17	¿Tiene algún problema con el movimiento de sus piernas, pies o rodillas?	No<br>Si<br>No especificado<br>No aplica	0<br>1<br>99<br>88	( ) Leve<br>( ) Moderado<br>( ) Grave	s750 extremidades inferiores (piernas, pies)
18	¿Tiene algún impedimento (Discapacidad / limitación)?	No<br>Si<br>No especificado<br>No aplica	0<br>1<br>99<br>88	( ) Leve<br>( ) Moderado<br>( ) Grave	-
19	En caso afirmativo, describa:	Visual<br>Audición<br>Motor<br>Mental o intelectual<br>Falta de estructuras especificar:________<br>Otro:________	0<br>1<br>2<br>3<br>4<br><br>5		-
20	Si no es así, ¿su enfermedad o afección causa limitaciones en sus actividades diarias o restricciones en su contacto con los demás?	No<br>Si<br>No especificado<br>No aplica	0<br>1<br>99<br>88	( ) Leve<br>( ) Moderado<br>( ) Grave	-
