# Supplementary material for: Validation of a cross-NTD toolkit for assessment of NTD-related morbidity and disability. A cross-cultural qualitative validation of study instruments in Colombia
Source: PLoS One. 2019 Dec 3;14(12):e0223042. doi: 10.1371/journal.pone.0223042 (PMC6890168; doi:10.1371/journal.pone.0223042)
Supplement: S2 Appendix — (PDF) [file pone.0223042.s006.pdf]

S2 Appendix. Clinical Profile English

CLINICAL PROFILE
NTD TOOLKIT – Body functions and structures
Participant ID number:______________ Diagnosis:________________ Clinical Profile:____________________

ITEM	QUESTION	CATEGORY		EXTENT OF IMPAIRMENT (when "YES")	ICF CODE AND CATEGORY
1.	Do you have any problem seeing things?	No<br>Yes<br>Not specified<br>Not applicable	0<br>1<br>99<br>88	( ) Mild<br>( ) Moderate<br>( ) Severe	b210 Seeing
2.	Do you have any problem hearing sounds or voices?	No<br>Yes<br>Not specified<br>Not applicable	0<br>1<br>99<br>88	( ) Mild<br>( ) Moderate<br>( ) Severe	b230 Hearing
3.	Do you have any problems with your skin? E.g. sensitivity or irritation	No<br>Yes<br>Not specified<br>Not applicable	0<br>1<br>99<br>88	( ) Mild<br>( ) Moderate<br>( ) Severe	b8. Functions of the skin and related structures
4.	Do you have any skin lesions?	No<br>Yes<br>Not specified<br>Not applicable	0<br>1<br>99<br>88	( ) Mild<br>( ) Moderate<br>( ) Severe	s8. Skin and related structures
5.	Do you have any open wounds?	No<br>Yes<br>Not specified<br>Not applicable	0<br>1<br>99<br>88	( ) Mild<br>( ) Moderate<br>( ) Severe	s8. Skin and related structures
6.	Do you experience pain in your chest and/or palpitations or are you easily tired?	No<br>Yes<br>Not specified<br>Not applicable	0<br>1<br>99<br>88	( ) Mild<br>( ) Moderate<br>( ) Severe	b410 Heart
7.	Do you have any problems breathing?	No<br>Yes<br>Not specified<br>Not applicable	0<br>1<br>99<br>88	( ) Mild<br>( ) Moderate<br>( ) Severe	b440 Respiration (breathing)
8.	Are you easily out of breath or do you have difficulty breathing?	No<br>Yes<br>Not specified<br>Not applicable	0<br>1<br>99<br>88	( ) Mild<br>( ) Moderate<br>( ) Severe	s430 Respiratory system
9.	Do you have any problems swallowing food? E.g. choking or food that gets stuck?	No<br>Yes<br>Not specified<br>Not applicable	0<br>1<br>99<br>88	( ) Mild<br>( ) Moderate<br>( ) Severe	b515 Digestion
10.	Do you have any problems with bowel	No	0	( ) Mild	b525

	movements or abnormal appearance of your stool? E.g. blood or worms	Yes<br>Not specified<br>Not applicable	1<br>99<br>88	( ) Moderate<br>( ) Severe	Defecation
11.	Do you pass too little urine or is there blood in your urine, or do you have pain when you try to pass urine?	No<br>Yes<br>Not specified<br>Not applicable	0<br>1<br>99<br>88	( ) Mild<br>( ) Moderate<br>( ) Severe	s610 Urinary system
12.	Do you have tremors, unusual movements, epileptic fits or problems controlling movements?	No<br>Yes<br>Not specified<br>Not applicable	0<br>1<br>99<br>88	( ) Mild<br>( ) Moderate<br>( ) Severe	s110 Brain
13.	Do you often experience pain?	No<br>Yes<br>Not specified<br>Not applicable	0<br>1<br>99<br>88	( ) Mild<br>( ) Moderate<br>( ) Severe	b280 Pain
14.	Do you experience pain, loss of feeling or weakness in your arms or legs?	No<br>Yes<br>Not specified<br>Not applicable	0<br>1<br>99<br>88	( ) Mild<br>( ) Moderate<br>( ) Severe	s120 Spinal cord and peripheral nerves
15.	Do you have any problems with insufficient strength in your arms or legs?	No<br>Yes<br>Not specified<br>Not applicable	0<br>1<br>99<br>88	( ) Mild<br>( ) Moderate<br>( ) Severe	b730 Muscle power
16.	Do you have any problems with movement of your arm, hand, wrist, elbows or shoulders?	No<br>Yes<br>Not specified<br>Not applicable	0<br>1<br>99<br>88	( ) Mild<br>( ) Moderate<br>( ) Severe	s730 Upper extremity (arm, hand)
17.	Do you have any problems with movement of your leg, foot or knees?	No<br>Yes<br>Not specified<br>Not applicable	0<br>1<br>99<br>88	( ) Mild<br>( ) Moderate<br>( ) Severe	s750 Lower extremity (leg, foot)
18.	Do you have an impairment (disability/limitation)?	No<br>Yes<br>Not specified<br>Not applicable	0<br>1<br>99<br>88	( ) Mild<br>( ) Moderate<br>( ) Severe	-
19.	If yes, please describe:	Visual<br>Hearing<br>Motor<br>Mental or intellectual<br>Absence of structure, specify:________<br>Other:________	0<br>1<br>2<br>3<br>4<br>5		-
20.	If not, does your disease or condition cause limitations in your daily activities or restrictions in your contact with others?	No<br>Yes<br>Not specified<br>Not applicable	0<br>1<br>99<br>88	( ) Mild<br>( ) Moderate<br>( ) Severe	-

NOTE: Questions 18 to 20 define the application (or not) of the WHOQOL-DIS.
Duration of interview:______ minutes
