# Supplementary material for: Validation of a cross-NTD toolkit for assessment of NTD-related morbidity and disability. A cross-cultural qualitative validation of study instruments in Colombia
Source: PLoS One. 2019 Dec 3;14(12):e0223042. doi: 10.1371/journal.pone.0223042 (PMC6890168; doi:10.1371/journal.pone.0223042)
Supplement: S3 Appendix — (PDF) [file pone.0223042.s007.pdf]

S3 Appendix. SRQ Spanish

SRQ (SELF REPORT QUESTIONNAIRE) - CUESTIONARIOS DE AUTO-RELATO

**DATOS PERSONALES**			
**NOMBRE:**			
**DIRECTRICES PARA LA REALIZACIÓN DE LA PRUEBA**			
Responda las siguientes preguntas acerca de su salud			
1. ¿Tiene dolores de cabeza frecuentes?	( ) Sí [1]	() NO [0]	
2. ¿Tiene falta de apetito?	( ) Sí [1]	() NO [0]	
3. ¿Duerme mal?	( ) Sí [1]	() NO [0]	
4. ¿Se asusta con facilidad?	( ) Sí [1]	() NO [0]	
5. ¿Tiene temblores en las manos?	( ) Sí [1]	() NO [0]	
6. ¿Se siente nervioso (a), tenso (a) o preocupado (a)?	( ) Sí [1]	() NO [0]	
7. ¿Tiene mala digestión?	( ) Sí [1]	() NO [0]	
8. ¿Tiene dificultad para pensar con claridad?	( ) Sí [1]	() NO [0]	
9. ¿Se ha sentido triste últimamente?	( ) Sí [1]	() NO [0]	
10. ¿Ha estado llorando más de lo habitual?	( ) Sí [1]	() NO [0]	
11. ¿Usted encuentra dificultades para realizar con satisfacción sus actividades diarias?	( ) Sí [1]	() NO [0]	
12. ¿Tiene problemas para tomar decisiones?	( ) Sí [1]	() NO [0]	
13. ¿Tiene dificultad en el servicio (su trabajo es dispendioso, le causa sufrimiento)?	( ) Sí [1]	() NO [0]	
14. ¿Es incapaz de desempeñar un papel útil en su vida?	( ) Sí [1]	() NO [0]	
15. ¿Ha perdido interés en las cosas?	( ) Sí [1]	() NO [0]	
16. ¿Se siente una persona inútil?	( ) Sí [1]	() NO [0]	
17. ¿Ha tenido pensamientos de acabar con su vida?	( ) Sí [1]	() NO [0]	
18. ¿Se siente cansado(a) todo el tiempo?	( ) Sí [1]	() NO [0]	
19. ¿Tiene sensaciones desagradables en el estómago?	( ) Sí [1]	() NO [0]	
20. ¿Se cansa con facilidad?	( ) Sí [1]	() NO [0]	
		**TOTAL**	
Nombre del responsable de aplicación del Test:			
Fecha:			

Breve descripción del SRQ (SELF REPORT QUESTIONNAIRE) - CUESTIONARIOS DE AUTO-RELATO

El SRQ es un cuestionario de identificación de los trastornos psiquiátricos en el nivel de atención primaria, que fue desarrollado por Harding et al. (1980) y validado en Brasil por MARI Y WILLIANS(1986).Consta de 20 preguntas diseñadas para detectar trastornos "neuróticos", llamados trastornos mentales actualmente comunes (TMC). Para que una persona pueda ser considerado como un posible caso se utilizan una puntuación de siete o más respuestas afirmativas (sí) por valor de un punto cada uno. Esta puntuación se obtuvo mediante la determinación de la sensibilidad, especificando valores predictivos positivos y negativos en otras dos muestras. Este punto de corte permite obtener dos grupos: por un lado los individuos con mayor probabilidad de tener un trastorno mental común y otro grupo con mayor probabilidad de no tenerlo.

Harding, T. W.; ARANGO, M.V.; BALTAZAR, J .; CLIMENT, C.E.; IBRAHIM, H.H.A.;IGNACIO, L.L.; MURTHY, RS Y PELUCA, NN (1980) - Trastornos Mentales en la atención primaria de salud: un estudio de su frecuencia y diagnóstico del desarrollo en cuatro países. Medicina Psicológica, 10: 231 a 241.

MARI, J. & WILLIAMS, PA. (1986).- El estudio de validez del cuestionario psiquiátrico de cribado (SRQ-20) en la atención primaria en la ciudad de São Paulo. Brit. J. Psychiatry, 148: 23-
