# Supplementary material for: Validation of a cross-NTD toolkit for assessment of NTD-related morbidity and disability. A cross-cultural qualitative validation of study instruments in Colombia
Source: PLoS One. 2019 Dec 3;14(12):e0223042. doi: 10.1371/journal.pone.0223042 (PMC6890168; doi:10.1371/journal.pone.0223042)
Supplement: S5 Appendix — (PDF) [file pone.0223042.s009.pdf]

S5 Appendix. WHOQOL-BREF & WHOQOL-DIS Spanish

WHOQOL-BREF (CORTO)

INSTRUCCIONES:

Este cuestionario es sobre cómo se siente acerca de su calidad de vida, la salud y otras áreas de su vida. Por favor, conteste todas las preguntas. Si no está seguro sobre qué respuesta dar a una pregunta, por favor elija la que parece más a propiada. Esto a menudo puede ser su primera opción.

Por favor, tenga en cuent a sus valores, aspiraciones, placeres y preo cupaciones. Le pedimos que usted piense en su vida, en referencia a las últimas dos semana s. Por ejemplo, teniendo en cuenta las últim as dos semanas, una pregunta podría ser:

	Nada	Muy poco	Medio	Mu cho	Totalmente
¿Recibe usted de otros el apoyo que necesita?	1	2	3	4	5

Usted debe rodear en un círculo el número que mejor corresponde c on la cantidad de apoyo que recibe de los demás en estas dos últimas semanas. Por lo tanto, usted debe rodear el número 4 si ha recibido “m ucho” apoyo.

	Nada	Muy poco	Medio	Mu cho	Totalmente
¿Recibe usted de otros el apoyo que necesita?	1	2	3	4	5

Usted debe rodear en un círculo el número 1, si usted no ha recibido "n ada" de apoyo.

Por favor, lea cada pregunta, según lo que piensa rodee en un círculo el número que le parece que la mejor respuesta.

	Muy malo	Malo	Ni malo ni bueno	Buena	Muy buena
1. ¿Cómo calificaría su calidad de vida?	1	2	3	4	5

	Muy insatisfecho	Insatisfecho	Ni satisfecho ni insatisfecho	Satisfecho	Muy satisfecho
2. ¿Qué tan satisfecho (a) está con su salud?	1	2	3	4	5

Las siguientes preguntas son acerca de las cosas que usted ha sentido en las últimas dos semanas.

	Nada	Muy poco	Más o menos	Bastante	Extremadamente
3. ¿En qué medida cree usted que su dolor (físico) le impide hacer lo que necesita?	1	2	3	4	5
4. ¿Usted necesita de algún tratamiento médico para desenvolverse en su vida diaria?	1	2	3	4	5
5. ¿Cuánto disfrutar usted de la vida?	1	2	3	4	5
6. ¿En qué medida siente usted que su vida tiene sentido?	1	2	3	4	5
7. ¿Cuánto poder de concentración tiene usted?	1	2	3	4	5
8. ¿Qué tan seguro (a) se siente en su vida diaria?	1	2	3	4	5
9. ¿Qué tan saludable es su entorno físico (clima, ruido, contaminación, atracciones)?	1	2	3	4	5

Las siguientes preguntas son acerca de las cosas que usted logro hacer completamente en las últimas dos semanas.

	Nada	Muy poco	Más o menos	Bastante	Extremadamente
10. ¿Tiene suficiente energía para su día a día?	1	2	3	4	5
11. ¿Es usted capaz de aceptar su apariencia física?	1	2	3	4	5
12. ¿Tiene suficiente dinero para satisfacer sus necesidades?	1	2	3	4	5

13. ¿Está disponible para usted la información que necesita en	1	2	3	4	5

su día a día?					
14. ¿Hasta qué punto tiene usted la oportunidad de realizar actividades de ocio?	1	2	3	4	5

Las siguientes preguntas son acerca de lo bueno o satisfecho que se ha sentido sobre diversos aspectos de su vida en las últimas dos semanas.

	Muy malo	Malo	Ni malo ni bueno	Buena	Muy buena
15. ¿Qué tan bien es usted capaz de moverse?	1	2	3	4	5
	Muy insatisfecho	Insatisfech o	Ni satisfecho ni insatisfecho	Satisfecho	Muy satisfecho
16. ¿Qué tan satisfecho (a) está con su sueño?	1	2	3	4	5
17. ¿Qué tan satisfecho (a) está con su capacidad de desempeño?	1	2	3	4	5
18. ¿Qué tan satisfecho (a) está usted con su capacidad para el trabajo?	1	2	3	4	5
19. ¿Qué tan satisfecho (a) está usted con usted mismo?	1	2	3	4	5
20. ¿Qué tan satisfecho (a) está en tus relaciones personales (amigos, familiares, conocidos, compañeros de trabajo)?	1	2	3	4	5
21. ¿Qué tan satisfecho (a) está con su vida sexual?	1	2	3	4	5
22. ¿Qué tan satisfecho (a) está usted con el apoyo que recibe de sus amigos?	1	2	3	4	5
23. ¿Qué tan satisfecho (a) está con las condiciones locales donde usted vive?	1	2	3	4	5
24. ¿Qué tan satisfecho (a) está con su acceso a los servicios de salud?	1	2	3	4	5
25. ¿Qué tan satisfecho (a) está con su transporte?	1	2	3	4	5

Las siguientes preguntas se refieren a la frecuencia con que ha sentido o experimentado ciertas cosas en las últimas dos semanas.

	Nunca	Algunas veces	A menudo	Muy a menudo	Siempre
26. ¿Con qué frecuencia tienen					

sentimientos negativos tales como mal humor, desesperación, la ansiedad, la depresión?	1	2	3	4	5

Alguien ayudó a llenar este cuestionario? ____

Cuánto tiempo le toma para completar este cuestionario? _________________________________

Tiene algún comentario sobre la encuesta? ____

MÓDULO DE DISCAPACIDADES

La siguiente pregunta es acerca de su discapacidad (discapacidad / limitación) en general.

		Nada	Muy poco	Normal	Mucho	Totalmente
27 G	¿Su discapacidad (discapacidad / limitación) tiene un efecto negativo (malo) en su vida diaria?	1	2	3	4	5

Las siguientes preguntas son acerca de cómo se sentía acerca de ciertas cosas, la forma en que se le aplicaron a usted y si está satisfecho con diversos aspectos de su vida en las últimas dos semanas.

		Nada	Muy poco	Normal	Mucho	Totalmente
28	¿Cree usted que algunas personas lo tratan injustamente?	1	2	3	4	5
29	¿Usted necesita que “alguien intervenga” cuando tiene problemas?	1	2	3	4	5
30	¿Le preocupa lo que pueda pasar en el futuro? Por ejemplo, pensando en no ser capaz de cuidar de sí mismo (a) o de ser una carga para otros en el futuro	1	2	3	4	5
31	¿Usted siente que controla su vida? Por ejemplo, usted se siente al mando de su vida?	1	2	3	4	5
32	¿Usted toma sus propias decisiones acerca de su vida en un día a día? Por ejemplo, acerca de dónde ir, qué hacer, qué comer	1	2	3	4	5
33	¿Usted toma las decisiones importantes en su vida? Por ejemplo, decidir dónde vivir, o	1	2	3	4	5

	con quién vivir, cómo gastar su dinero.					

34	¿Está usted satisfecho (a) con su capacidad para comunicarse con los demás? Por ejemplo, cómo dice las cosas y defiende su punto de vista, o de qué modo usted entiende a otras personas a través de palabras o signos.	☺ 1	2	😐 3	4	☹ 5
35	¿Usted se siente aceptado por otras personas?	1	2	3	4	5

		Nada	Muy poco	Normal	Mucho	Totalmente
36	¿Cree usted que otras personas lo respetan?<br>Por ejemplo, usted siente que los demás lo valoran como persona y escuchan lo que tiene que decirles?	1	2	3	4	5
37	¿Está usted satisfecho (a) con sus posibilidades de involucrarse en actividades sociales?<br>Por ejemplo, para encontrar amigos, salir a comer fuera, ir a una fiesta, etc.	1	2	3	4	5
38	¿Está usted satisfecho (a) con sus posibilidades de participar en las actividades de su comunidad (local)?<br>Por ejemplo, participar en lo que está sucediendo en su localidad o barrio.	1	2	3	4	5
39	¿Siente que sus sueños, expectativas y deseos se harán realidad? Por ejemplo, usted siente que tiene la oportunidad de hacer las cosas que quiere o conseguir las cosas que quiere en su vida.	1	2	3	4	5

¿Tiene algún comentario acerca de esta encuesta?

………
…………………………………………………

………
…………………………………………………

………
…………………………………………………

………
……………………….

GRACIAS POR SU AYUDA

☺ 😐 ☹
