# Supplementary material for: Validation of a cross-NTD toolkit for assessment of NTD-related morbidity and disability. A cross-cultural qualitative validation of study instruments in Colombia
Source: PLoS One. 2019 Dec 3;14(12):e0223042. doi: 10.1371/journal.pone.0223042 (PMC6890168; doi:10.1371/journal.pone.0223042)
Supplement: S6 Appendix — (PDF) [file pone.0223042.s010.pdf]

S6 Appendix. WHOQOL-BREF & WHOQOL-DIS English

WHOQOL-BREF and WHOQOL-DIS
**NTD TOOLKIT – Personal factors**
Participant ID number:_____________

WHOQOL-BREF

Please think about your life **in the last two weeks:** The first two questions ask about your life and health overall.

	Very poor	Poor	Neither poor nor good	Good	Very good
1G. How would you rate your quality of life?	1	2	3	4	5

	Very satisfied	Dissatisfied	Neither satisfied nor dissatisfied	Satisfied	Very satisfied
2G. How satisfied are you with your health?	1	2	3	4	5

The following questions ask about how much you have experienced certain things in the last two weeks.

	Not at all	A little	A moderate amount	Very much	An extreme amount
3. To what extent do you feel that (physical) pain prevents you from doing what you need to do?	1	2	3	4	5
4. How much do you need any medical treatment to function in your daily life?	1	2	3	4	5
5. How much do you enjoy life?	1	2	3	4	5
6. To what extent do you feel your life to be meaningful?	1	2	3	4	5

	Not at all	A little	A moderate amount	Very much	Extremely
7. How well are you able to concentrate?	1	2	3	4	5
8. How safe do you feel in your daily life?	1	2	3	4	5
9. How healthy is your physical environment?	1	2	3	4	5

The following questions ask about **how completely** you experienced or were able to do certain things in the last two weeks.

	Not at all	A little	Moderatel y	Mostly	Completely
10. Do you have enough energy for everyday life?	1	2	3	4	5
11. Are you able to accept your bodily appearance?	1	2	3	4	5
12. Have you enough money to meet your needs?	1	2	3	4	5
13. How available to you is the information that you need in your day-to-day life?	1	2	3	4	5
14. To what extent do you have the opportunity for leisure activities?	1	2	3	4	5

	Very poor	Poor	Neither poor nor good	Good	Very good
15. How well are you able to get around?	1	2	3	4	5

The following questions ask you to say how **good or satisfied** you have felt about various aspects of your life over the **last two weeks.**

	Very dissatisfied	Dissatisfied	Neither satisfied nor dissatisfied	Satisfied	Very satisfied
16. How satisfied are you with your sleep?	1	2	3	4	5
17. How satisfied are you with your ability to perform your daily living activities?	1	2	3	4	5

18. How satisfied are you with your capacity for work?	1	2	3	4	5
19. How satisfied are you with yourself?	1	2	3	4	5
20. How satisfied are you with your personal relationships?	1	2	3	4	5
21. How satisfied are you with your sex life?	1	2	3	4	5
22. How satisfied are you with the support you get from your friends?	1	2	3	4	5
23. How satisfied are you with the conditions of your living place?	1	2	3	4	5
24. How satisfied are you with your access to health services?	1	2	3	4	5
25. How satisfied are you with your transport?	1	2	3	4	5

The following question refers to how often you have felt or experienced certain things in the last two weeks.

	**Never**	**Seldom**	**Quite often**	**Very often**	**Always**
26. How often do you have negative feelings such as blue mood, despair, anxiety, depression?	1	2	3	4	5

Duration of interview:______ minutes

WHOQOL-DIS

The next question asks about your disability overall.

	Not at all	A little	Moderately	Mostly	Totally
27G. Does your disability have a negative (bad) effect on your day-to-day life?	1	2	3	4	5

The following questions ask about how you have felt about certain things, how much certain things have applied to you, and how satisfied you have been about various parts of your life over the last two weeks.

	Not at all	A little	Moderately	Mostly	Totally
28. Do you feel that some people treat you unfairly?	1	2	3	4	5
29. Do you need someone to stand up for you when you have problems?	1	2	3	4	5
30. Do you worry about what might happen to you in the future?<br><br>*For example, thinking about not being able to look after yourself, or being a burden to others in the future.*	1	2	3	4	5
31. Do you feel in control of your life?<br><br>*For example, do you feel in charge of your life?*	1	2	3	4	5
32. Do you make your own choices about your day-to-day life?<br><br>*For example, where to go, what to do, what to eat.*	1	2	3	4	5
33. Do you get to make the big decisions in your life?<br><br>*For example, like deciding where to live, or who to live with, how to spend your money.*	1	2	3	4	5
34. Are you satisfied with your ability to communicate with other people?<br><br>*For example, how you say things or get your point across, the way you understand others, by words or signs.*	1	2	3	4	5
35. Do you feel that other people accept you?	1	2	3	4	5

36. Do you feel that other people respect you?<br><br>*For example, do you feel that others value you as a person and listen to what you have to say?*	1	2	3	4	5

	**Not at all**	**A little**	**Moderately**	**Mostly**	**Totally**
37. Are you satisfied with your chances to be involved in social activities?<br><br>*For example, meeting friends, going out for a meal, going to a party etc.*	1	2	3	4	5
38. Are you satisfied with your chances to be involved in local activities?<br><br>*For example, being part of what is happening in your local area or neighbourhood.*	1	2	3	4	5
39. Do you feel that your dreams, hopes and wishes will happen?<br><br>*For example, do you feel you will get the chance to do the things you want, or get the things you wish for, in your life?*	1	2	3	4	5

Do you have any comments about the questionnaire?

____

____

____

____
