# Supplementary material for: Validation of a cross-NTD toolkit for assessment of NTD-related morbidity and disability. A cross-cultural qualitative validation of study instruments in Colombia
Source: PLoS One. 2019 Dec 3;14(12):e0223042. doi: 10.1371/journal.pone.0223042 (PMC6890168; doi:10.1371/journal.pone.0223042)
Supplement: S7 Appendix — (PDF) [file pone.0223042.s011.pdf]

S7 Appendix. Interview guide Spanish

Parte 1. Introducción
Antes de iniciar la entrevista usted desea establecer una buena relación con el participante. Por lo tanto,

- Dar las gracias al participante para participar en este estudio
- Explique quién es usted / quiénes somos y cuál es el objetivo de la investigación
- Explicar cómo se utilizarán los datos
- Explicar que la participación es voluntaria; si el participante no quiere responder a una pregunta o quiere detener la entrevista está su libre elección para hacerlo
- Pídale al participante que firme el consentimiento informado antes de continuar la conversación
- Preguntar si podemos grabar la conversación
- Preguntarle si tiene algún comentario o pregunta

Parte 2. Administración de las herramientas

- Todas las preguntas del cuestionario serán formuladas exactamente como están escritas en el formulario
- Durante la administración de los cuestionarios notas serán hechas para cada pregunta:
  Y, Si una pregunta es directamente entendida,
  X, Si una pregunta tiene que ser reformulada antes de que se entienda, y
  O, Si se necesitan dar ejemplos para que la pregunta sea entendida.

- *Esta parte de la entrevista evaluará la equivalencia semántica debido a su entendimiento, aunque no en profundidad. Además, el entrevistador es capaz de observar si el participante se encuentra a gusto con el modo de administración y si entienden directamente las escalas para responder a las preguntas. Esto evaluará la equivalencia operacional.*

Parte 3. Evaluación de las equivalencias
Después de la aplicación de cada instrumento se le preguntará al participante su pensamiento sobre el cuestionario. La siguiente parte de la entrevista consta de preguntas abiertas. Pregunte a los participantes si pueden explicar con más detalle sus respuestas para obtener una mayor comprensión en profundidad (sondeo).

Parte 3.1. Evaluación conceptual e ítem equivalente

- ¿Cuáles son sus pensamientos acerca de la relevancia del cuestionario para su situación particular?
- ¿Cuáles son sus pensamientos acerca de la pertinencia de las preguntas separadas en este cuestionario?
  Para esta pregunta se puede hacer referencia a los signos antes de cada pregunta durante la administración del cuestionario, véase la parte 2 de la entrevista

Parte 3.2. Evaluación de la equivalencia semántica
¿Cree que las preguntas del cuestionario fueron bien formuladas? (se entienden)
Si no es así, cuáles preguntas?
¿Cómo pueden las palabras o preguntas ser cambiadas para que sean más claras?
¿Cree que las palabras utilizadas en las preguntas son comprensibles para la comunidad colombiana en general?

Parte 3.3. Evaluación de la equivalencia operativa

- ¿Cuáles son sus pensamientos acerca de las posibilidades de respuesta utilizados para responder a las preguntas?
- ¿Habían palabras, frases o preguntas que le hicieron sentir incómodo?
    - Si es así, ¿cuál?
    - ¿Por qué le hizo sentir incómodo?
    - ¿Cómo se pueden cambiar para que no lo hagan sentir incómodo?

Parte 4. Experiencia general del participante

Después de aplicar todas las herramientas hay algunas preguntas generales. Además, se quiere expresar gratitud.

- ¿Encontró la duración total de esta entrevista aceptable? ¿Por qué si o por qué no?
- ¿Cuál fue la impresión general que de esta entrevista? ¿Positivo o negativo?
- ¿Tiene algún otro comentario / comentarios o preguntas sobre la entrevista en este momento?

- La entrevista ha finalizado. ¡Muchas gracias por tu tiempo! Si usted tiene alguna pregunta sobre la entrevista no dude en ponerse en contacto con nosotros. Agradecemos su disposición a participar en este estudio!
