# Supplementary material for: Validation of a cross-NTD toolkit for assessment of NTD-related morbidity and disability. A cross-cultural qualitative validation of study instruments in Colombia
Source: PLoS One. 2019 Dec 3;14(12):e0223042. doi: 10.1371/journal.pone.0223042 (PMC6890168; doi:10.1371/journal.pone.0223042)
Supplement: S8 Appendix — (PDF) [file pone.0223042.s012.pdf]

S8 Appendix. Interview guide English

Part 1. Introduction

Before starting the interview you want to establish a good relationship with the participant. Therefore,

- Thank the participant for participating in this study
- Explain who you/we are and the aim of the research
- Explain how the data will be used
- Explain participation is voluntary; if the participant does not want to answer a question or want to stop the interview it is their free choice to do so
- Ask the participant to sign the informed consent before continuing the conversation
- Ask if we can record the conversation
- Ask for any comments or questions

Part 2. Administration of the tools

- All questions of the questionnaire will be asked exactly as written on the form
- During the administration of the questionnaires notes will be made for each question:
  ✓ if a question is directly understood,
  ✗ if a question needs to be reformulated before the question is understood, and **O** if examples need to be given before a question is understood.

- *This part of the interview will assess semantic equivalence because of their understanding although not in-depth. Furthermore the interviewer is able to observe if the participant is at ease with the mode of administration and if they directly understand the scales to answer the questions. This will assess the operational equivalence.*

Part 3. Assessment of the equivalences

After applying each instrument the participant will be asked about their thought of the questionnaire. The following part of the interview consists of open questions. Ask the participants if they can further explain their answers to gain a more in-depth understanding (probing questions).

Part 3.1. Assessment of the conceptual and item equivalence

- What are your thoughts about the relevance of the questionnaire to your particular situation?
- What are your thoughts about the relevance of the separate questions in this questionnaire?

  For this question you can refer to the signs before each question during administration of the questionnaire, see part 2 of the interview

Part 3.2. Assessment of the semantic equivalence

- Do you think the questions in the questionnaire were well formulated? (able to understand)
  - If not, which questions?
  - How can the words or questions be changed so you would understand the questions?
  - Do you think the words used in the questions are understandable for the general Colombian people?

Part 3.3. Assessment of the operational equivalence

- What are your thoughts about the answer possibilities used to answer the questions?
- Were there any words, phrases or questions that made you feel uncomfortable?
  - If yes, which one?
  - Why did it make you feel uncomfortable?
  - How can these be changed so it does not make you feel uncomfortable?

Part 4. Overall experience of the participant

After applying all tools there are some overall questions. Furthermore, you want to express gratitude.

- Did you find the overall duration of this interview acceptable? Why or why not?
- What was you overall impression of this interview? Positive or negative?
- Do you have any other remarks/comments or questions about the interview at the moment?

- The interview is now finished. Thank you very much for your time! If you have any questions about the interview do not hesitate to contact us. We appreciate your willingness to participate in this pilot study!
