# Supplementary material for: Validation of a cross-NTD toolkit for assessment of NTD-related morbidity and disability. A cross-cultural qualitative validation of study instruments in Colombia
Source: PLoS One. 2019 Dec 3;14(12):e0223042. doi: 10.1371/journal.pone.0223042 (PMC6890168; doi:10.1371/journal.pone.0223042)
Supplement: S9 Appendix — (PDF) [file pone.0223042.s013.pdf]

S9. Focus group guide Spanish

Guía reunión grupos focales (participantes)

Antes de comenzar la reunión del grupo focal, dar la bienvenida a todos los participantes y las gracias por su tiempo y participación en el estudio. Todos han participado en las entrevistas y son conscientes de quiénes somos y del objetivo de la investigación. Es útil repetirlo brevemente. Asegúrese de que todos los participantes hayan firmado el formulario de consentimiento informado.

Bienvenida:

Bienvenidos a todos y gracias por ofrecerse a participar en este grupo de discusión. Les hemos pedido a participar, ya que su punto de vista es importante. Apreciamos su tiempo.

Somos (dar nombres) y estamos trabajando en conjunto con la Universidad Metropolitana, DAHW, y la VU-Universidad.

El objetivo de este estudio es validar las herramientas incluidas el kit de herramientas NTD, mediante el análisis de estas herramientas con las personas afectadas por la lepra, la leishmaniasis o las enfermedades tropicales desatendidas NTD en Colombia. (NTD explicar si esto no está claro)

Durante esta reunión del grupo seleccionado nos gustaría obtener su opinión en profundidad acerca de los cuestionarios. La reunión del grupo focal tendrá una duración de no más de 2 horas.

Anonimato:

Debido al valor de todas sus opiniones, nos gustaría grabar la voz de la discusión. Estas grabaciones se mantendrán a salvo hasta que se transcriban, y a continuación, se destruirán. En estas transcripciones ninguna información personal será procesada, por ejemplo, no se utilizarán nombres. ¿Está bien para todos? (En caso afirmativo, iniciar grabación)

Nos gustaría pedirle que responda a las preguntas lo más exactamente posible y con la verdad, no hay respuestas incorrectas

Si no desea responder a una pregunta o participar de un cierto debate, usted es libre de hacerlo. Aunque, su opinión es muy valorada.

Reglas básicas: Explique brevemente algunas reglas básicas:

Usted no tiene que hablar en un orden particular

Una persona habla a la vez, por favor espere hasta que alguien haya terminado No hay respuestas correctas o incorrectas

Usted no tiene que estar de acuerdo con las opiniones de los miembros del grupo

Pregunte si hay alguna pregunta hasta el momento?

Calentamiento: En primer lugar, nos gustaría que cada uno se presente. Esto hace que sea más fácil hablar durante el debate. Para personalizar, trate de preguntar acerca de una experiencia/hecho positivo.

En primer lugar, nos gustaría a todos se presenten. Para llegar a conocerse un poco, nos gustaría saber de dónde es y lo que más le gusta de su ciudad / municipio.

Gusto en conocerlos a todos, nos gustaría iniciar la discusión acerca del Kit de herramientas ahora.

Guía de preguntas: Esta parte se centrará en cada herramienta separada. El orden de las herramientas no importa; esto puede cambiar cada grupo de discusión. Palabras clave pueden ser escritas en los papeles y se coloca al lado de la herramienta el comentario. Tomar una foto de la colección puede ser útil para su uso posterior.

Primera parte:

¿Cuáles son sus pensamientos acerca de la relevancia del cuestionario a su situación particular?

¿Cuáles son sus pensamientos acerca de la relevancia de las preguntas separadas en este cuestionario?

¿Fue capaz de entender todas las preguntas en el cuestionario?

- Si no es así, cuáles preguntas?
- ¿Cómo pueden cambiar las palabras o preguntas para que sea entendido?
- ¿En qué medida cree usted que el nivel de español en el cuestionario es apropiado?

¿Cuáles son sus pensamientos acerca de las escalas utilizadas para responder a las preguntas?

¿Había palabras, frases o preguntas que le hicieron sentir incómodo?

- Si es así, ¿cuál?
- ¿Por qué le hizo sentir incómodo?
- ¿Cómo éstas pueden ser cambiadas para que no le hagan sentir incómodo?

Segunda parte:

¿Podría describir cómo su NTD influye en su vida diaria?

¿Podría describir si hay personas en su entorno, por ejemplo, la familia o amigos, que le ayuden con las condiciones provocadas por la Lepra en su vida diaria?

¿Recibe el apoyo de una institución de salud pública o servicios?

- En caso afirmativo, ¿podría describir cómo contribuyen las instituciones públicas o servicios de salud?
  - medicación
  - Terapia; mental o física
  - Herramientas; por ejemplo, sillas de ruedas o muletas o ...?

Con las preguntas hemos intentado crear un panorama general de su vida diaria con Lepra / Leishmaniasis

- ¿Podría describir cómo cree que las preguntas representan su vida diaria?

Podría describir si hay aspectos de los servicios de salud pública que podrían cambiar para que reciba más apoyo y tal vez podría disfrutar más la vida?

Preguntas finales:

¿Cuál fue que la impresión general del kit de herramientas NTD ¿Positivo o negativo?

¿Encontró aceptable la duración total del grupo de discusión? ¿Por qué si o por qué no?

¿Tiene algún otro comentario o preguntas acerca del kit de herramientas NTD en este momento?

Conclusión:

Gracias a todos por participar. Este ha sido un debate muy exitoso. Sus opiniones serán un activo valioso para el estudio

Esperamos que haya encontrado interesante la discusión

Si tiene alguna pregunta o comentario más tarde, póngase en contacto con nosotros (dar información de contacto)

Nos gustaría recordarle que todos los comentarios que ofrecen este informe son anónimos

Se le invita a tomar una bebida (ubicación). ¡Que tengas un buen día!
