# Supplementary material for: Validation of a cross-NTD toolkit for assessment of NTD-related morbidity and disability. A cross-cultural qualitative validation of study instruments in Colombia
Source: PLoS One. 2019 Dec 3;14(12):e0223042. doi: 10.1371/journal.pone.0223042 (PMC6890168; doi:10.1371/journal.pone.0223042)
Supplement: S10 Appendix — (PDF) [file pone.0223042.s014.pdf]

S10 Appendix. Focus group guide English

Focus group meeting guide (participants)

Before starting the focus group meeting, welcome all the participants and thank them for their time and participation in the study. All these participants have been involved in the interviews and are aware of who we are and what the aim of the research is. It is useful to briefly repeat this. Make sure all participants have signed the informed consent form.

Welcome:

Welcome everyone and thank you for volunteering to take part in this focus group. You have been asked to participate, as your point of view is important. We appreciate your time.

We are (names) and are working together with Universidad Metropolitana, DAHW,and the VU-University.

The aim of this study is to validate the tools included in the cross-NTD toolkit, by testing these tools with individuals affected by the NTDs leprosy or leishmaniasis in the NTD-endemic country Colombia. (explain NTD if this is unclear)

During this focus group meeting we would like to obtain your in-depth opinion about the questionnaires. The focus group meeting will last no more than 2 hours.

Anonymity:

Because we value all your opinions, we would like to voice record the discussion. These recordings will be kept safe until they are transcribed, then they will be destroyed. In these transcriptions no personal information will be processed, for example no names will be used. Is that okay with everyone? (If yes, press record)

We would like to ask you to answer the questions as accurate and truthfully as possible, there are no wrong answers

If you do not want to answer a question or participate a certain discussion, you are free to do so. Although, your opinion is highly valued

Ground rules: Briefly explain some ground rules:

You do not have to speak in a particular order

One person speaks at a time, please wait until someone has finished

There are no right or wrong answers

You do not have to agree with the views of the group members

Ask if there are any questions so far?

Warming up: First, try to let everyone introduce him or herself. This makes it easier to speak up during the discussion. To personalize this, try to ask them about a positive experience/fact. (favourite fruits and colour)

First, we would like everyone to introduce him or herself. To get to know each other a little, we would like to know where you are from and what you like best about your city/town.

Nice to meet you everyone, we would like to start the discussion about the tools of the toolkit now.

Guiding questions: This part will focus one each tool separate. The order of the tools does not matter; this can change every focus group. Key words can be written down on papers and placed next to the tool the comment is about. Taking a picture of the collection can be useful for further use.

Part one:

What are your thoughts about the relevance of the questionnaire to your particular situation?

What are your thoughts about the relevance of the separate questions in this questionnaire?

Were you able to understand all the questions in the questionnaire?

- If not, which questions?
- How can the words or questions be changed so you would understand the questions?
- To what extend do you think the level of Spanish in the questionnaire is appropriate?

What are your thoughts about the scales used to answer the questions?

Were there any words, phrases or questions that made you feel uncomfortable?

- If yes, which one?
- Why did it make you feel uncomfortable?
- How can these be changed so it does not make you feel uncomfortable?

Which questionnaire did you like best, which was the hardest, and why?

Part two:

Could you describe how your NTD influences your daily life?

Could you describe if there are people in your environment, for example family or friends, that help you with conditions resulted by Lepra in your daily life?

Do you receive any support from a public health care institution or services?

- If yes, could you describe how public health care institutions or services contribute?
  - Medication
  - Therapy; mental or physical
  - Tools; for example wheelchair or crutches or …?

With the questions we have tried to create an overview of your daily life living with Lepra/Leishmaniasis

- Could you describe how you think the questions represent your daily life?

Could you describe if there are aspects of the public health care services which could change so you receive more support and could maybe enjoy life more?

Concluding questions:

What was you overall impression of the NTD-toolkit? Positive or negative?

Did you find the overall duration of the focus group acceptable? Why or why not?

Do you have any other remarks/comments or questions about the NTD-toolkit at the moment?

Conclusion:

Thank you all for participating. This has been a very successful discussion. Your opinions will be a valuable asset to the study

We hope you have found the discussion interesting

If you have any questions or comments later on, please contact us (give contact information)
We would like to remind you all comments featuring this report will be anonymous
You are invited to have a drink (location). Have a nice day!
