# Supplementary material for: Validation of a cross-NTD toolkit for assessment of NTD-related morbidity and disability. A cross-cultural qualitative validation of study instruments in Colombia
Source: PLoS One. 2019 Dec 3;14(12):e0223042. doi: 10.1371/journal.pone.0223042 (PMC6890168; doi:10.1371/journal.pone.0223042)
Supplement: S11 Appendix — (PDF) [file pone.0223042.s015.pdf]

S11 Appendix. Informed Consent

CONSENTIMIENTO INFORMADO

Lugar y Fecha: ____

Yo, ____
Identificado con C.C. ___________________ de ____________ declaro que he sido informado(a) por: _________________________________ acerca de la investigación denominada EVALUACIÓN Y SEGUIMIENTO DE MORBILIDAD Y DISCAPACIDAD RELACIONADA CON LAS ENFERMEDADES TROPICALES DESATENDIDAS: HERRAMIENTA GENERAL DE DESARROLLO EN COLOMBIA, que he comprendido el propósito del estudio, que he tenido oportunidad de aclarar mis dudas, que estoy satisfecho(a) con la información proporcionada y que de forma libre y voluntaria acepto contestar las encuestas que obtendrán información para la misma.

Este documento ha sido leído y entendido por mí en su integridad.

Firma: __________________________________ C.C.
