# Supplementary material for: Validation of a cross-NTD toolkit for assessment of NTD-related morbidity and disability. A cross-cultural qualitative validation of study instruments in Colombia
Source: PLoS One. 2019 Dec 3;14(12):e0223042. doi: 10.1371/journal.pone.0223042 (PMC6890168; doi:10.1371/journal.pone.0223042)
Supplement: S12 Appendix — (PDF) [file pone.0223042.s016.pdf]

S12 Appendix. COREQ-checklist and Research group.

Consolidated criteria for reporting qualitative studies (COREQ): 32-item checklist.

No. Item	Guide questions/description	Reported on Page #
**Domain 1: Research team and reflexivity**		
Personal Characteristics		
1. Inter viewer/facilitator	Which author/s conducted the inter view or focus group?	Page 12
2. Credentials	What were the researcher's credentials? E.g. PhD, MD	Appendix 12
3. Occupation	What was their occupation at the time of the study?	Appendix 12
4. Gender	Was the researcher male or female?	Appendix 12
5. Experience and training	What experience or training did the researcher have?	Page 12
Relationship with participants		
6. Relationship established	Was a relationship established prior to study commencement?	S7-10
7. Participant knowledge of the interviewer	What did the participants know about the researcher? e.g. personal goals, reasons for doing the research	Page 13 and S7-8
8. Interviewer characteristics	What characteristics were reported about the inter viewer/facilitator? e.g. Bias, assumptions, reasons and interests in the research topic	Page 12

**Domain 2: study design**		
Theoretical framework		
9. Methodological orientation and Theory	What methodological orientation was stated to underpin the study? e.g. grounded theory, discourse analysis, ethnography, phenomenology, content analysis	Page 8
Participant selection		
10. Sampling	How were participants selected? e.g. purposive, convenience, consecutive, snowball	Page 6
11. Method of approach	How were participants approached? e.g. face-to-face, telephone, mail, email	Page 6
12. Sample size	How many participants were in the study?	Page 6
13. Non-participation	How many people refused to participate or dropped out? Reasons?	Page 35
Setting		
14. Setting of data collection	Where was the data collected? e.g. home, clinic, workplace	Page 12
15. Presence of non-participants	Was anyone else present besides the participants and researchers?	No
16. Description of sample	What are the important characteristics of the sample? e.g. demographic data, date	Page 6<br>S1 Table
Data collection		
17. Interview guide	Were questions, prompts, guides provided by the authors? Was it pilot tested?	Appendix 7-8
18. Repeat interviews	Were repeat inter views carried out? If yes, how many?	x
19. Audio/visual recording	Did the research use audio or visual recording to collect the data?	Page 12
20. Field notes	Were field notes made during and/or after the inter view or focus group?	Page 12
21. Duration	What was the duration of the inter views or focus group?	Page 28

22. Data saturation	Was data saturation discussed?	Page 6
23. Transcripts returned	Were transcripts returned to participants for comment and/or correction?	x
**Domain 3: analysis and findings**		
Data analysis		
24. Number of data coders	How many data coders coded the data?	Page 2
25. Description of the coding tree	Did authors provide a description of the coding tree?	no
26. Derivation of themes	Were themes identified in advance or derived from the data?	no
27. Software	What software, if applicable, was used to manage the data?	Page 13
28. Participant checking	Did participants provide feedback on the findings?	no
Reporting		
29. Quotations presented	Were participant quotations presented to illustrate the themes/findings? Was each quotation identified? e.g. participant number	yes
30. Data and findings consistent	Was there consistency between the data presented and the findings?	yes
31. Clarity of major themes	Were major themes clearly presented in the findings?	yes
32. Clarity of minor themes	Is there a description of diverse cases or discussion of minor themes?	yes

Tong A, Sainsbury P, Craig J. Consolidated criteria for reporting qualitative research (COREQ): a 32-item checklist for interviews and focus groups. *International Journal for Quality in Health Care*. 2007. Volume 19, Number 6: pp. 349 – 357

Researchers	Credentials	Occupation	Gender	Experiences
Janneke Fischer	BSc	Master student Biomedical Sciences, VU-University Amsterdam	Female	Medium
Benita Jansen	BSc	Master student International Public Health, VU-University Amsterdam	Female	Medium
Alberto Rivera	MD PhD	Head of South American Affaires of the DAHW	Male	High
Libardo J. Gómez	MD	Coordinator at the DAHW, Medical consultant	Male	High
Martha C. Barbosa	-	Social worker at the DAHW, research supporter	Female	High
Jorge L. Bilbao	MD	Head of Faculty of Health Sciences at University Metropolitana, Barranquilla	Male	High
José M. González	BSc	Master student Medicine at University Metropolitana, Barranquilla	Male	Low
Luis Restrepo	BSc	Master student Medicine at University Metropolitana, Barranquilla	Male	Low
Yesenia Vidal	PhD	PhD, head of Molecular Biology at University Metropolitana, Barranquilla	Female	High
Ruth M. H. Peters	PhD	Postdoc and lecturer at VU University, Amsterdam	Female	High
